# Public health action on extreme heat in three Canadian provinces: a scoping review

**DOI:** 10.3389/phrs.2026.1609625

**Published:** 2026-07-31

**Authors:** Mélanie S. S. Seabrook, Imaan Umar, Stephanie Simpson, Ana Patricia Ayala, Edward C. Xie, Q. Jane Zhao, Sara Allin

**Affiliations:** 1 Institute of Health Policy, Management, and Evaluation, Dalla Lana School of Public Health, University of Toronto, Toronto, ON, Canada; 2 North American Observatory on Health Systems and Policies, Toronto, ON, Canada; 3 Gerstein Science Information Centre, University of Toronto, Toronto, ON, Canada; 4 Department of Family and Community Medicine, University of Toronto, Toronto, ON, Canada; 5 Division of Emergency Medicine, University Health Network, Toronto, ON, Canada; 6 Upstream Lab, MAP Centre for Urban Solutions, Li Ka Shing Knowledge Institute, Unity Health Toronto, Toronto, ON, Canada; 7 Project ECHO, Toronto Rehabilitation Institute, Toronto, ON, Canada

**Keywords:** Canada, climate change, extreme heat, heat response, public health

## Abstract

**Objectives:**

To examine how public health activities addressing extreme heat align with Canada’s Essential Public Health Functions; the roles taken by public health authorities in collaborative responses; and the extent of heat response evaluations to date in three Canadian provinces.

**Methods:**

Following Arksey and O’Malley’s Framework, we conducted a scoping review of academic and grey literature on public health heat strategies in British Columbia, Ontario, and Quebec, Canada between 2005 and 2026. We searched ten academic databases, and scanned government and non-government websites. Academic sources were double-screened using Covidence software.

**Results:**

We identified 26 peer-reviewed articles (British Columbia: 7, Ontario: 8, and Quebec: 16) and 429 grey literature sources. Nine sources evaluated heat responses across the three provinces. Public health authorities across the provinces fulfilled similar Essential Public Health Functions, focused on downstream response including establishing heat alert systems and communicating preventative health information. Public health actors primarily play advisory roles in intersectoral heat responses, though some co-lead responses.

**Conclusion:**

Our findings suggest ongoing gaps in upstream public health heat strategies, and meaningful collaborations with at-risk communities.

## Introduction

The frequency and intensity of worldwide extreme heat events are expected to increase as a consequence of climate change throughout the 21st century [1]. Extreme heat exposure contributes to excess morbidity and mortality through health impacts including heat stroke, dehydration, cardiovascular and respiratory disease, and negative mental health effects [2]. Models estimate extreme heat causes 489,000 excess deaths per year world-wide [3].

In Canada, population centres in southern and coastal regions experience extreme heat, with related mortality rising sharply over the past decade [4]. Most recently, the 2018 Quebec heatwave caused 80 deaths [5], and the 2021 “Heat Dome” in Western Canada resulted in 619 deaths [6]. High risk populations include older adults, children, individuals with chronic physical or mental health conditions, low-income households, and those experiencing homelessness [7].

Given the escalating health risks, public health authorities have an imperative to mitigate the health impacts of climate change. Public health activities addressing heat range Essential Public Health Functions (EPHFs), offered by Canada’s Chief Public Health Officer to describe and categorize the breadth and depth of public health work, and are described in Table 1 [2, 8–12]. Public health leaders and researchers have also called for greater collaboration between public health authorities and other sectors (e.g., housing, environment, and community services) to effectively address the factors shaping heat vulnerability [2, 12, 13].

**TABLE 1:** Heat activities aligned with essential public health functions in Canada (2026).

Essential public health function	Definition	Heat activities
Health promotion	Improving healthy public policy, community-based interventions, public participation, advocacy or action on determinants of health	Greening infrastructure
Health surveillance	Collecting health data for disease tracking and monitoring health inequities	Tracking heat illness and mortality
Health protection	Protecting the population from infectious diseases, environmental threats, and unsafe food, water, and air	Education on actions to prevent heat illness
Population health assessment	Conducting research to understand the health of communities and populations, determinants of health, and identifying interventions	Heat vulnerability assessments
Disease and injury prevention	Promoting healthy behaviours to prevent illness and injury	Community-based heat adaptations
Emergency prediction, preparedness, and response	Planning for natural or human-made disasters and responding to emergencies	Heat alert and response systems, health check-ins, cooling spaces, heat response personnel training

The Essential Public Health Functions are defined by the Canada’s Chief Public Health Officer’s 2022 report on mobilizing public health action for climate change [2].

In Canada, public health action on extreme heat spans national and sub-national levels [14]. At the national level, two government institutions–the Public Health Agency of Canada and Health Canada‐both provide leadership around broad public health aims and functions [15], whereas Health Canada also takes a lead role in extreme heat [16]. Sub-nationally, public health authorities vary in how they engage with environmental health threats like extreme heat [7, 17–20]. This variation has yet to be investigated and documented in the academic literature, though a recent government-funded report examined Heat Alert and Response System implementation across Canada [21].

This scoping review examines the approach and activities of public health authorities in preventing extreme heat health impacts in the three most populous provinces in Canada–Ontario, Quebec, and British Columbia (BC). Specifically, we report how public health heat activities align with EPHFs, and characterize the roles of public health authorities, including leadership, advisory, and coordinating roles, within collaborations. We also report the extent to which public health heat strategies have been evaluated.

## Methods

We conducted a scoping review of academic and grey literature to examine the extent and nature of existing provincial, regional, and local extreme heat strategies in BC, Ontario, and Quebec. Our study was guided by the Arksey and O’Malley Scoping Review Framework and reported according to the PRISMA-ScR Checklist [22, 23]. The search strategy was developed by an academic health sciences librarian (AA) (see Supplementary Data Sheet 1). Nine academic databases were searched: Ovid MEDLINE, Ovid CAB, EBSCO GreenFile, ProQuest PAIS, ProQuest Policy File, Engineering Village GEOREF, Engineering Village GEOBASE, Web of Science Core Collection, and Scopus. Search strategies were adapted to each database’s command language and appropriate search fields. MeSH terms and textwords captured concepts related to public health, extreme heat, and the three provinces for the original search in Ovid MEDLINE. The search was peer reviewed by a second librarian using PRESS [24] before being translated to other databases. Searches were limited by date (January 1, 2005, and June 19, 2026) based on severe heatwaves triggering new responses in 2004, and language (English and French).

Academic citations were uploaded to Covidence, an online software facilitating article sorting and screening. Sources were screened by individual reviewer pairs (MS, IU, SS) following the inclusion criteria in Table 2, starting with title and abstracts, followed by full-text review. Disagreements were resolved through discussion.

**TABLE 2:** Inclusion criteria for academic and grey literature (scoping review, Ontario, British Columbia, and Quebec, Canada, 2005–2026).

Criteria	Include
Geography	British Columbia, Ontario, Quebec
Units of analysis	Local, regional, provincial
Focus	Must address public health approach to addressing impacts of extreme or chronic heat exposure
Type of intervention	Prevention, mitigation, preparedness, adaptation, response, recovery, resilience
Date range	2005–2026
Languages	French and English
Format	Journal articles, editorials, reports, public government documents, legislation, infographics/interactive tools/video, webpages

Grey literature was identified through targeted scans of government, public health agency, and non-governmental organization websites in the three provinces. Scans were supplemented by targeted Google searches using a combination of terms (e.g., [public health authority name] + “extreme heat”) if no results were identified from public health authority websites. Sources were included based on the same inclusion criteria as the academic literature (Table 2). Original searches were completed on November 18, 2023, and a targeted academic and grey literature search update, including Google searches of heat response initiatives undertaken by all three provinces’ public health authorities, was completed on June 24, 2026.

### Data extraction and analysis

Data from included sources were extracted according to the research objectives, using data extraction templates (Supplementary Data Sheet 2). The EPHFs described in Canada’s Chief Public Health Officer’s 2022 report on mobilizing public health action for climate change were applied as a framework to report on the range of public health heat activities identified (Table 1) [2]. Narrative synthesis methods were used to characterize and compare public health roles in addressing extreme heat in each province, and to report on any evaluations of heat response activities.

## Results

### Review sources

The academic literature search yielded 6370 results (after duplicate removal), of which 26 articles were included for analysis (Supplementary Data Sheet 3: PRISMA diagram). Sixteen articles focused on extreme heat response in Quebec, eight in Ontario, and seven in BC (Table 3, and see Supplementary Data Sheet 4 for details). Of these, three articles focused on two or more provinces [25–27].

**TABLE 3:** Overview of types of academic articles included (scoping review, Ontario, British Columbia, and Quebec, Canada, 2005–2026).

Study type	Ontario	British Columbia	Quebec
Descriptive reviews of heat strategies or interventions	5	1	7
Epidemiological reports	0	2	1
Methodological development for establishing heat warning thresholds	0	0	2
Evaluations	1	1	3
Governance analysis	1	2	2
Content analysis	1	2	1
TOTAL	8	7	16

Four distinct articles are counted more than once: a descriptive article that appears under both Ontario and Quebec; a governance analysis article and a content analysis article that each appear under Ontario, BC, and Quebec; and a BC article that is counted twice because it uses both evaluation and governance analysis methods.

A total of 429 grey literature sources were retrieved across BC (n = 132), Ontario (n = 167), and Quebec (n = 130). These included informational text and graphic resources via webpages, reports (e.g., vulnerability assessments), protocols and guides, policy documents (e.g., legislation), and interactive tools such as heat maps, educational videos, and infographics. Publicly accessible heat strategies were limited: we identified 6 regional health system heat plans in Quebec, 8 municipal heat plans with public health involvement in Ontario, and 5 municipal heat plans with health authority involvement in BC.

### Overview of public health heat activities in each province

The following sections detail how public health authorities in each province have developed and exercise their roles in responding to extreme heat. Provincial governments set standards and fund public health services, while public health units, departments, and agencies administer the programs.

#### Ontario

Ontario’s public health system consists of 29 local public health units (PHUs) as of 2026, and a provincial public health agency (Public Health Ontario) [19]. The Ontario Public Health Standards, pursuant to the *Health Protection and Promotion Act* [28], establish minimum requirements for PHU program delivery. Although these requirements are intentionally broad to allow programs to be tailored to local context, they require PHUs to “prevent and reduce the burden of illness from […] extreme weather and extreme temperatures” [29]. Specific to heat, PHUs are expected, but not mandated, to: 1) receive and communicate Environment and Climate Change Canada (ECCC, a federal department that monitors and predicts extreme weather) heat warnings; 2) review and share guidance with local partners; and 3) conduct surveillance of heat health impacts and evaluate heat response activities [29]. The reviewed literature suggests variation in how PHUs meet these expectations.

Regarding the first expectation, and aligned with the *emergency prediction, preparedness and response,* and *health protection* EPHFs, the province’s Harmonized Heat Warning and Information System communicates heat warnings to the public. The system, co-developed in 2016 by ECCC, Health Canada, the Ontario Ministry of Health, Public Health Ontario, and partnered PHUs [30], is incorporated into the Ontario Public Health Standards, and addresses prior variations in heat alert and response systems across PHUs [29, 31]. Specifically, it standardizes thresholds for issuing local heat warnings across the province, with ECCC issuing warnings to all affected PHUs based on regional temperature and humidex criteria. Most PHUs display ECCC heat warnings and information on heat-related illnesses and locations of cooling centres and hydration stations on their websites.

Regarding the second expectation, and aligned with the *emergency prediction, preparedness and response* EPHF, PHUs vary in how they review and share heat response guidance; some lead heat response plans and interventions [32–34] whereas others appear to play more supporting and advisory roles to municipal authorities [35–39]. The provincial Office of the Chief Medical Officer of Health also advises PHUs on heat emergency planning [29].

Regarding the third expectation on surveillance and evaluation, and aligned with the *population health assessment* EPHF, PHUs can use the Ontario Climate Change and Health Toolkit developed by the provincial Ministry of Health in 2016 to assess health vulnerabilities within their communities [30]. Several PHUs have led population heat health impact and vulnerability assessments that are publicly available on their websites [40–43].

#### British Columbia

BC public health services are delivered through five regional health authorities (RHAs) and one First Nations Health Authority [20]. The BC Centre for Disease Control (BCCDC) provides public health-related analytical and policy support to RHAs, and conducts population health research and surveillance [20]. After the 2021 “Heat Dome”, the BC Ministry of Health and BCCDC established the BC Health Effects of Anomalous Temperatures Committee (BC HEAT Committee) to coordinate extreme heat response across levels of governance [44]. BC’s *Public Health Act (2008)* and *Emergency and Disaster Management Act (2023)* authorize provincial ministries, RHAs, and public health officials to lead and respond to heat emergency events [44]. As part of the *Climate Preparedness and Adaptation Strategy*, the province developed an extreme heat preparedness guide for use by provincial government agencies, and expanded the Community Emergency Preparedness Fund to include a dedicated stream for local governments and First Nations communities to prepare and respond to extreme heat events [45].

Aligned with the *emergency prediction, preparedness and response* and *health protection* EPHFs, the BC HEAT Committee established a two-tiered provincial heat alert and response system (HARS) in 2022. Updated annually, the HARS consists of standardized, province-wide temperature thresholds that trigger “heat warnings” and “extreme heat emergencies” that in turn mobilize regional emergency management efforts [46]. It also includes recommended actions for government sectors, RHAs (including public health divisions), hospitals and community care sites, Indigenous Governing Bodies, Non-Governmental Organizations (NGOs), and partner organizations [46]. Information on heat health protective actions are also published on provincial and RHA webpages [47]. Relatedly, the BCCDC published a guide in 2017 for municipalities seeking to develop heat response plans, including recommendations on collaborations with regional public health divisions [48]. RHAs also provide guidelines for municipal implementation of cooling centres [27].

Aligned with the *population health assessment* and *health surveillance* EPHFs, the BCCDC and RHAs also conduct research and surveillance on heat-related mortality to generate policy-relevant evidence [49–51]. For example, evidence of socioeconomic disparities in mortality risk during the 2021 “Heat Dome” has since led to greater emphasis on social, economic, and built environment factors in government communications and health authority response planning [50]. Furthermore, two RHAs co-led a climate vulnerability assessment in 2022, including population-level and health system impacts from extreme heat [49].

#### Quebec

Quebec was the first Canadian province to develop an extreme heat response plan, driven by public health initiative following significant mortality caused by heatwaves in 2001 and 2002 [25, 52, 53]. Currently, the *Loi sur la Santé Publique* gives public health authorities special authority to protect population health from threats such as extreme heat [54–56]. Quebec’s provincial public health programme, *Programme National de Santé Publique*, further specifies that public health authorities are responsible for sharing preventative heat health information, conducting risk assessments, surveillance and epidemiological reporting, providing protective targeted interventions, implementing alert systems, and providing preparedness, intervention, and recovery services related to extreme heat [57].

Quebec’s public health institute, *Institut National de Santé Publique du Québec* (INSPQ), plays advisory, coordinative, and evaluative roles in addressing the health effects of extreme heat. As subject-matter experts, the INSPQ has produced knowledge syntheses and heat adaptation guides for public health authorities [58, 59]. As part of Quebec’s *2006–2012 Action Plan on Climate Change*, the Ministry of Health & Social Services (MSSS) mandated the INSPQ to create a surveillance and warning system for heatwaves [60–62] and evaluate programs aimed at reducing urban heat islands [60, 62, 63].

The INSPQ surveillance and warning system, known as SUPREME, fulfils the *health surveillance* EPHF. Its functions are to: 1) compile extreme weather data and health impact indicators; 2) transmit real-time extreme weather alerts to personnel; and 3) host a Geoportal website that maps data on a broad range of risks and resources to inform climate planning [64], including mapping urban heat islands [53, 65]. Every year, INSPQ publishes a surveillance report on the health impacts of heatwaves [66, 67].

Other heat-related activities led by provincial public health authorities align with the *health promotion* and *emergency prediction, preparedness and response* EPHFs. Specifically, the INSPQ funded 40 urban heat island reduction pilot projects implemented by municipalities and NGOs through the provincial Green Fund financed by the Quebec carbon tax [62, 63]. The INSPQ also conducts epidemiological analyses of the health impacts of heat waves [68], and helps develop evidence-based thresholds for Quebec’s heat warning system [69, 70]. The provincial public health department (*Direction Générale de la Santé Publique*) monitors heat indicators and co-coordinates the MSSS extreme heat management plan to support regional public health responses [54].

Regional public health activities reflect *health surveillance, health protection, population health assessment,* and *emergency prediction, preparedness and response* EPHFs. Quebec’s public health services are regionalized through 22 public health departments integrated within 18 health and social service regions [17], and the MSSS heat management plan requires each to have up-to-date heat response plans [54]. Regional public health departments are responsible for activating regional heat alerts, public prevention communications, and coordinating with health system emergency units, municipal governments, and NGOs as part of regional heat response plans [26, 54]. They also provide a variety of advisory resources including epidemiological reports on severe heatwaves [63–66, 71–74], local vulnerability assessments [75–81], and heat response guides targeted to municipalities, health services, and other public and private entities [67, 68, 82, 83].

Overall, despite some variations in the legislative frameworks in place, public health authorities primarily perform advisory roles in the three provinces (described in Table 4). Exceptions include the BCCDC and BC Ministry of Health co-leading the BC HEAT Committee, and instances of provincial and regional public health co-leadership in Quebec. In the following sections, we report public health roles in collaborative actions, followed by heat response evaluations across the three provinces.

**TABLE 4:** Public health heat response across Ontario, British Columbia, and Quebec, Canada (2005–2026).

Province	Legislated mandate and powers of public health authorities in heat response	Provincial heat alert system	Provincial public health role in heat response strategy	Regional/Local public health role in heat response strategy
Ontario	The Ontario Public Health Standards (pursuant to Section 7 of the *Health Protection and Promotion Act)* establishes minimum requirements for PHU services, including to reduce the burden of illness from extreme temperatures [29]	Harmonized Heat Warning and Information System (HWIS): Heat warnings issued by ECCC initiate heat response plans led by municipalities and PHUs [30]	Advisory role: *The Office of the Chief Medical Officer of Health, Public Health:* Advises PHUs regarding heat emergency planning and evaluation activities [29] *Public Health Ontario:* Conducts epidemiological analyses and evidence reviews to inform heat strategy implementation and improvements [30, 31, 84, 85]	Supporting role (with some exceptions): Most PHUs play supporting roles in their municipal/regional heat response strategies (e.g., communicating heat warnings) [29]. Some PHUs lead or co-lead heat response strategies [32–34]
British Columbia	The *Public Health Act* and the *Emergency and Disaster Management Act* authorize public health officials and RHAs to lead and respond to heat emergency events, but does not mandate specific responses [44]	Heat Alert and Response System (HARS): Threshold-specific heat warnings, issued by ECCC, and extreme heat emergencies, issued through coordination between the BC HEAT operations sub-committee and ECCC. Both tiers are accompanied by distinct recommended actions for various government sectors and partner organizations [46]	Lead role: *BCCDC* Works with RHAs, ECCC and health Canada to establish heat alert thresholds, leads the BC HEAT committee alongside the Ministry of Health, and conducts surveillance of heat-related health impacts [46, 50]	Supporting role: Most public health departments within RHAs have advisory roles in local heat response strategies (e.g., implementing heat response communications) [46, 86]
Quebec	The *Loi sur la Santé Publique* gives public health authorities special powers to protect population health under threat [56]. Quebec’s *Programme National de Santé Publique,* (established within the *Loi sur la Santé Publique* legal framework), specifies public health responsibilities for addressing environmental threats including extreme heat [57]	*Système de Surveillance et de Prévention des impacts sanitaires des Evènements Météorologiques Extrêmes* (SUPREME): Issues heat alerts to regional public health departments who decide whether to activate the alert level of their plans (imminent threat). If they activate, the MSSS also activate to the alert level of the provincial plan [54] If multiple regions activate the alert level at the same time, the MSSS activates their full plan to coordinate across regions [54] If extreme heat threshold is met, the regional public health department decides to escalate to the mobilization (emergency) level response. Thresholds differ by region [54]	Lead role: *Institut National de la Santé Publique du Québec (INSPQ):* Hosts and manages the SUPREME weather surveillance and heat alert system [53] *Direction Générale de la Santé Publique:* Co-coordinates the provincial heat response strategy for actors within the health and social services sector. Mobilizes provincial heat response strategy when two or more regions activate heat alerts to align regional responses [54]	Central role in health and social sectors response: Regional public health departments are responsible for activating regional heat alerts, public prevention communications, and coordinating with health system emergency units, municipal governments, and NGOs as part of regional heat response plans [54]

### Public health roles in collaborations addressing extreme heat

Our review identified collaborative efforts to address extreme heat across organizations (horizontal collaboration), across levels of government (vertical collaboration), and across sectors (intersectoral collaboration). BC was the only province with a clear framework facilitating horizontal intersectoral collaboration at the provincial level through the BC HEAT committee, co-led by the BCCDC and BC Ministry of Health. Across all provinces, collaboration at local and regional levels was more common than at the provincial level, often spanning public health authorities, local governments, and other sectors and partner organizations (e.g., community organizations). Municipalities often lead these collaborations as part of their heat response plans, with public health authorities playing an advisory role [26, 37, 86, 87]. Examples of public health’s collaborative leadership include the Waterloo Region Extreme Heat and Cold Partnership [33] and North Bay Parry Sound District Heath Unit’s Extreme Heat Advisory Plan [34] in Ontario. Additionally, the Northern Ontario Climate Change and Health collaborative is a unique public health-specific network of seven PHUs seeking to collaboratively enhance their capacities to mitigate climate change health impacts [88].

In Quebec, many local public health departments co-lead heat response collaboration between regional and municipal civil security divisions [74, 83, 89]. For example, the Montreal regional public health department collaborates closely with the health system emergency unit (*Coordination régionale des mesures d’urgence, de la sécurité civile et accès réseau*) and the City of Montreal’s Civil Security Centre to plan population-level interventions and ensure alignment between the health system and the city heat response plans [26, 55]. Some public health authorities were observed to coordinate NGO partnerships to establish green spaces [90, 87], and to support seniors and individuals with mental health conditions measure indoor air temperatures [91].

Our review also identified examples of ‘vertical’ public health collaboration. Health Canada, the Public Health Agency of Canada, and the INSPQ regularly support the climate adaptation efforts of local public health authorities through expertise and *ad hoc* funding [92]. Notably, Health Canada finances multiple provincial and regional public health authorities conducting climate change health vulnerability assessments and heat response planning through the HealthADAPT program [49, 80, 92–94], and the recent HeatADAPT program [95].

### Extent of existing evaluations of heat responses

Our review did not uncover any comprehensive evaluations of provincial or sub-provincial heat response strategies. Existing evaluations focus on specific response components or outcomes. Six evaluations were identified in Quebec, two in Ontario, and one in BC. Those in Quebec pertain to Montreal’s local heat response plan and provincial systems related to extreme weather. A 2007 evaluation of a public heat health education campaign by Montreal’s Public Health Department revealed a positive association between preventative messaging and adoption of preventative measures during heat events. The evaluation also highlighted that limited informational, financial, and social resources impeded the ability of high-risk seniors to adopt these measures [96]. A 2018 study analyzing the implementation of the Montreal heat response plan similarly found that while preventative measures were known and applied by healthcare personnel, their awareness and application was variable among vulnerable populations [97]. The study also highlighted the need to rapidly mobilize sufficient personnel as a central challenge of responding to heatwaves [97]. A related study from 2018 examined the validity of the term “vulnerable populations” in the Montreal heat response plan, finding that while individuals with schizophrenia do tend to view themselves as more vulnerable to heat, people with drug or alcohol addictions may not. The study also revealed that certain heat response interventions, such as targeted phone check-ins, were not always well received and could benefit from co-design with affected communities [98]. A 2016 quantitative evaluation found that the Montreal heat response plan helped to reduce mortality on hot days, with larger effects among elderly and low-socioeconomic status populations [99].

In 2017, an evaluation of the sensitivity and specificity of SUPREME in predicting heatwaves concluded that, while the system performed well, its sensitivity could be improved through minor adjustments to its alert criteria [100]. Finally, a 2016 evaluation of a selection of the urban heat island reduction projects funded by INSPQ found that some achieved significant cooling effects [63].

In Ontario, a population-based time series analysis of the impacts of the provincial Harmonized Heat Warning and Information System between 2012-2018 revealed a reduction in emergency department visits among certain populations, including those under 18 years of age, with co-morbidities, or living in rural communities, though the changes were not statistically significant [101]. Furthermore, a 2012 qualitative study on climate change adaptation efforts identified the need for provincial public health standards that explicitly address extreme heat mitigation and adaptation goals, and increased federal and provincial funding to support local adaptation strategies [43]. Study participants stated the importance of intersectoral collaboration to implement adaptation activities, such as multi-agency workshops to conduct extreme heat vulnerability assessments.

A 2025 BC study evaluated cooling centres across municipalities in Greater Vancouver through semi-structured interviews with public sector professionals [27]. Identified challenges included limited funding, staffing shortages, and infrastructure constraints. Opportunities to strengthen planning and operations include better collaboration across sectors and NGOs, improved outreach to communities, and harmonization of extreme weather responses.

## Discussion

This scoping review is, to our knowledge, the first comparing public health actions in heat responses across multiple Canadian provinces, and complements a recent government-funded report on HARS implementation in Canada [21]. Specifically, we aimed to report how public health heat activities across Ontario, BC, and Quebec are aligned with EPHFs, characterize the roles taken by public health authorities, and report the extent to which public health heat strategies have been evaluated. We uncovered limited attention within the academic literature to public health action in Ontario and BC; most studies were from Quebec, perhaps owing to the dedicated funding for public health research on extreme heat through the provincial Green Fund, as well as Quebec’s long-standing public health focus on addressing extreme heat [26, 61, 102]. The grey literature provided a wide range of sources demonstrating public health actions addressing extreme heat across the three provinces, however publicly accessible heat strategies were limited.

We found that public health authorities across all three provinces are largely engaged in activities related to *emergency prediction, preparedness and response, health protection,* and *population health assessment*. Core public health activities consisted of establishing province-wide heat alert systems, conducting heat vulnerability assessments, and publicly communicating preventative heat health information. One study that examined 99 public health authority websites across Canada found that Ontario accounted for 37% of all communication related to heat health information, with BC at 23% and Quebec at 10% [103]. Epidemiologic research activities on heat-related health impacts were also conducted by public health authorities in BC and Quebec, relating to the *health surveillance* EPHF. Notably, we identified articles or reports of activities relating to the *health promotion* EPHF only in Quebec, specifically through the urban heat island reduction pilot projects, and identified no clear instances of *disease and injury prevention*.

The three provinces’ public health heat activities are similar to other jurisdictions including the United States and Europe [8–10]. Yet, the extent to which public health authorities engage in upstream prevention of health risks was unclear, such as developing and advocating for indoor temperature bylaws, air conditioning access programs, tenant protections, and urban heat island mitigation [21, 61, 63], to complement the apparent focus on downstream preparedness and response [2, 12]. Public health activities that span the upstream-downstream continuum are necessary to effectively address climate-related health impacts [2]. Relatedly, we found limited evidence of public health engagement with communities, particularly those most at risk of health impacts from heat. Supporting community-based adaptation and mitigation strategies enables context-specific approaches that leverage local knowledge and leadership [2, 10].

Our search identified that all three provinces have legislation in place that mandates public health authorities to protect populations from health threats and authorizes special powers for response. However, as these legislative frameworks do not stipulate actions on extreme heat, public health responsibilities vary. Our review also identified similarities and differences across the three provinces in terms of how public health authorities’ roles in heat response are characterized. At the provincial level, the BCCDC and BC Ministry of Health co-lead the intersectoral BC HEAT Committee, while Quebec’s provincial public health authorities, the *Direction Générale de la Santé Publique* and INSPQ, help coordinate health and social service sector response, with particular support for public health interventions. In Ontario, we identified an advisory role for the Office of the Chief Medical Officer of Health and Public Health Ontario. We found that sub-provincial public health authorities play an advisory role in heat response strategies in BC, whereas in Quebec and Ontario, some regional heat response plans suggest co-leadership responsibilities for their public health departments [26].

Intersectoral collaboration on extreme heat response was prominent across the three provinces, and some studies have examined the role of public health authorities in the intersectoral governance of heat strategies. Two studies found that formal governance arrangements supporting extreme heat response are concentrated between the provincial and regional levels rather than with the federal government [26, 92]. Furthermore, legislative mandates, formal response plans outlining roles and responsibilities, and coordinating structures for network engagement supported effective intersectoral collaboration on extreme heat response in Canadian cities and reduced duplication of efforts [26, 27, 92]. Intersectoral governance analyses may help inform more equitable strategies for mobilizing resources and expertise from diverse sectors.

### Core challenges and facilitators in addressing extreme heat

Actors face multiple challenges in responding to extreme heat, including emergency workforce availability, identifying priority populations, sustained funding, political support, and effective communications [26, 96, 97, 104]. Recent studies have also highlighted ongoing gaps in governance to align work on climate emergencies [26, 104, 105]. Nevertheless, provincial guides and toolkits, vulnerability mapping, and cross-jurisdictional learning seem to support development and improvement of heat responses [26, 58, 65]. Targeted funds such as Quebec’s Green Fund also enabled the implementation of effective preventative measures such as urban greening projects and research supporting the advancement of heat strategies [59, 102]. BC’s extreme heat-targeted funding through the Community Emergency Preparedness Fund was initiated to support local governments and First Nations communities in extreme heat risk mapping and response efforts [106]. However, we could not uncover specific examples of how communities used this funding.

Published heat strategy evaluations remain limited in Canada. Our review found no comprehensive evaluation of provincial or sub-provincial heat strategies, and a related report found few HARS include an evaluation plan in Canada [21]. Existing evaluations focused on a single intervention or outcome of interest. This reflects a broader global trend of under-evaluation of heat responses [10]. Nonetheless, quantitative strategy evaluations identified in this review reported reductions in heat mortality and emergency department visits, for vulnerable populations in particular, and qualitative studies made recommendations for improvement of heat strategy components. Strategy evaluations are important for assessing both the effectiveness and quality of interventions in producing health outcomes, though collecting outcomes data and establishing causal links to heat strategy interventions remains challenging [10, 11]. However, strategies may be evaluated without relying on core measures and causal links, such as collecting data on alternative explanatory factors, and leveraging incremental plan changes to evaluate the impacts of new interventions [107]. Researchers could support more comprehensive evaluations, and partnerships across agencies can facilitate data sharing [107].

Furthermore, vulnerability to extreme heat is higher in certain populations depending on various biological, psychological, and socioeconomic factors [7]. Our review found that public health authorities across the three provinces conduct vulnerability assessments to identify populations at high risk to extreme heat, and convey information targeted to these populations. For example, public health authorities developed webpages and infographics specific to children, seniors, and workers [103, 108–112], and conducted targeted phone check-ins with at-risk individuals [98]. In this way, public health response to extreme heat aims to reduce inequitable heat health impacts. Nevertheless, one study demonstrated that groups identified as “vulnerable to heat” by public health authorities do not necessarily perceive themselves as such, and that targeted interventions are not always well-received [98]. This highlights the need for improved engagement of diverse populations in local heat response planning to prevent stigmatization and develop appropriate interventions [13]. Other studies have recommended greater efforts towards policy change to improve material and social conditions, reflecting broader calls to prevent heat and climate vulnerability by addressing socioeconomic and political structures as the primary drivers of vulnerability and inequitable heat impacts [13, 113]. International perspectives also support moving towards a collective approach to heat resilience and public health equity through community-based approaches and stronger intersectoral collaboration [11, 13, 113]. Moving toward such system-level approaches in close collaboration with high-risk communities could help mitigate the inequitable health effects caused by heatwaves by reorienting strategies toward addressing the root causes of vulnerability.

### Limitations

There are some limitations to this study. While nearly all public health authorities scanned provide extreme heat health information resources, many do not publish heat response plans, making it difficult to gauge the full extent of work being done. As a result, we reported public health responsibilities as outlined by provincial agencies, but were not always able to verify whether these are put into practice. Publishing public health strategies and plans would facilitate knowledge sharing across regions, as well as further research supporting continued strategy improvement [107]. We minimized the risk of reporting bias, a prevalent limitation of scoping reviews [114], by establishing data extraction templates that were used in all three cases (Supplementary Data Sheet 2).

### Conclusion

This scoping review highlights how public health authorities across three Canadian provinces tend to play an advisory role within heat responses, though some are involved in coordination of efforts across sectors. While public health authorities appear primarily focused on downstream responsive heat-related activities, some public health leaders propose how the public health sector may also be critical to advancing upstream prevention and resilience. Our findings suggest there is room for public health authorities to further clarify and develop their role in addressing extreme heat impacts, including building meaningful collaborations with communities and across sectors.
