# Supplementary material for: Public health action on extreme heat in three Canadian provinces: a scoping review
Source: Public Health Rev. 2026 Jul 31;47:1609625. doi: 10.3389/phrs.2026.1609625 (PMC13473856; doi:10.3389/phrs.2026.1609625)
Supplement: Supplementary file 1 [file DataSheet1.pdf]

Supplementary File 1: Academic search strategy

Ovid MEDLINE

Database(s): **Ovid MEDLINE: Epub Ahead of Print, In-Process & Other Non-Indexed Citations, Ovid MEDLINE® Daily and Ovid MEDLINE®** 1946-Present
Search Strategy:

#	Searches	Results
1	carbon cycle/ or carbon sequestration/ or climate change/ or global warming/ or droughts/ or El Nino-Southern Oscillation/ or floods/ or greenhouse effect/ or Hot Temperature/ or ("Arctic amplification" or "Arctic shrinkage" or "carbon footprint" or "carbon offset*" or "carbon sequestration" or "carbon sink" or "carbon sinks" or (chlorofluorocarbon* adj2 (release or concentration* or atmosphere*)) or (climate adj3 (change* or model?ing or predict* or resilience or sensitivity or action* or variability or adaptation or crisis or breakdown)) or deglaciation or desertification or "natural disaster*" or heatwave* or drought or droughts or heat-dome* or (earth adj2 warming) or El Nino or La Nina or "emissions reduc*" or "emissions trading" or flood* or high-temp* or ((glacial or glacier) adj3 (retreat* or melt*)) or "global radiation*" or "global temperature**" or "global warming" or "greenhouse effect" or "greenhouse gas*" or "heat wave**" or heatwave* or hurricane** or "ice cap melt*" or "ice mass loss*" or "Kyoto Protocol" or "landslide*" or "land slide*" or "mudslide*" or "mud slide*" or (ozone adj2 hole) or "Paris Accord" or "permafrost melt*" or "polar amplification" or "polar ice melt*" or "sea ice shrinkage" or "sea level rise*" or "sea surface warming" or "storm surge*" or "thermohaline circulation" or ((extreme or severe or destructive) adj3 (temperature* or weather* or storm* or hail or wind* or climate or heat or heating or warm* or humidity)) or tornado* or typhoon* or wildfire* or "wild fire*").mp.	359417
2	(flood* or hurricane* or polar amplification or sea level rise* or sea surface warming or storm surge or wild fire* or wildfire*).tw,kf.	32673
3	((increasing or rising or increase* or extreme or "rise in") adj3 (humidity or humid or temperature* or weather* or heat* or hot* or warm*)).tw,kf.	90941
4	(heat* adj4 (stroke* or hazard* or expos* or wave* or dome or chronic* or prolong* or event* or warn* or shock* or stress* or alert* or alarm*)).tw,kf.	86434
5	(thermal adj3 (expos* or hazard*)).tw,kf.	2010
6	(heatshock* or hyperthermia* or "heat shock*" or heat-shock).tw,kf.	85287
7	((global* or planet* or earth) adj3 (heating or warming)).tw,kf.	13962
8	Extreme Heat/	589
9	Hot Temperature/	125608
10	Temperature/	265568
11	Climate/	25867
12	limit 11 to yr="1993 - 2009"	7211
13	Heat-Shock Response/	9477
14	public health/ or public health practice/	101540
15	public policy/ or environmental policy/ or health policy/ or policy making/	119541
16	(collab* adj4 (govern* or action* or approach* or policy or policies or response*)).tw,kf.	11324
17	government/ or government agencies/ or local government/ or state government/	44620
18	((intersection* or inter-section* or intersector* or multi-sector* or inter-sector* or multisector* or cross-sector* or inter-department* or interdepartment* or transdisciplin* or trans-disciplin*) adj4 (approach* or collab* or partner* or action* or response*)).tw,kf.	6928
19	(environ* adj5 (health* or safety)).tw,kf.	80566

20	((public* or region* or local* or city or cities or municipal* or provincial* or province* or territor* or town* or communit*) adj5 (govern* or health* or policy or policies)).tw,kf.	596378
21	british columbia/ or ontario/ or quebec/	58120
22	(British Columbia or Colombie Britannique or Ontario or Quebec*).mp.	88114
23	(Abbotsford or Ajax or Aurora or Barrie or Belleville or Blainville or Brampton or Brantford or Brossard or Burlington or Burnaby or Caledon or Chatham Kent or Chilliwack or Clarington or Coquitlam or Drummondville or Gatineau or Granby or Sudbury or Guelph or Halton Hills or Kamloops or Kawartha Lakes or Kelowna or Kingston or Kitchener or Langley or Laval or Levis or Longueuil or Maple Ridge or Markham or Mirabel or Mississauga or Montreal or New Westminster or Newmarket or Niagara Falls or Norfolk County or North Bay or North Vancouver or Oakville or Oshawa or Ottawa or Peterborough or Pickering or Port Coquitlam or Prince George or Quebec City or Repentigny or Saanich or Saguenay or Saint-Hyacinthe or Saint-Jean-sur-Richelieu or Saint-Jerome or Sarnia or Sault Ste Marie or Sherbrooke or St Catharines or Surrey or Terrebonne or Thunder Bay or Toronto or Trois-Rivieres or Vancouver or Vaughan).tw,kw.	63159
24	((Halifax or Hamilton or London or Victoria or Waterloo or Welland or Whitby or Windsor or Richmond or Milton) not (UK or Britain or United Kingdom or England or Australia or New Zealand)).tw,kw.	57086
25	1 or 2 or 3 or 4 or 5 or 6 or 7 or 8 or 9 or 10 or 12 or 13	724479
26	14 or 15 or 16 or 17 or 18 or 19 or 20	821104
27	21 or 22 or 23 or 24	192079
28	25 and 26 and 27	303
29	limit 28 to yr="2005 - 2023"	287
30	**limit 29 to (english or french)**	**286**

Ovid CAB Abstracts

Database(s): **CAB Abstracts** 1973 to 2023 Week 46
Search Strategy:

#	Searches	Results
1	("Arctic amplification" or "Arctic shrinkage" or "carbon footprint" or "carbon offset*" or "carbon sequestration" or "carbon sink" or "carbon sinks" or (chlorofluorocarbon* adj2 (release or concentration* or atmosphere*)) or (climate adj3 (change* or model?ing or predict* or resilience or sensitivity or action* or variability or adaptation or crisis or breakdown)) or deglaciation or desertification or "natural disaster*" or heatwave* or drought or droughts or heat-dome* or (earth adj2 warming) or El Nino or La Nina or "emissions reduc*" or "emissions trading" or flood* or ((glacial or glacier) adj3 (retreat* or melt*)) or "global radiation*" or "global temperature**" or "global warming" or "greenhouse effect" or "greenhouse gas*" or "heat wave**" or heatwave* or hurricane** or "ice cap melt*" or "ice mass loss*" or "Kyoto Protocol" or "landslide*" or "land slide*" or "mudslide*" or "mud slide*" or high-temp* or (ozone adj2 hole) or "Paris Accord" or "permafrost melt*" or "polar amplification" or "polar ice melt*" or "sea ice shrinkage" or "sea level rise*" or "sea surface warming" or "storm surge*" or "thermohaline circulation" or ((extreme or severe or destructive) adj3 (temperature* or weather* or storm* or hail or wind* or climate or heat or heating or warm* or humidity)) or tornado* or typhoon* or wildfire* or "wild fire*").mp.	573418
2	(flood* or hurricane* or polar amplification or sea level rise* or sea surface warming or storm surge or wild fire* or wildfire*).tw,hw.	121099
3	(heat-shock* or hyperthermia* or heatschok* or "heat shock*").tw,hw.	23656
4	((increasing or rising or increase* or extreme or "rise in") adj3 (humidity or humid or temperature* or weather* or heat* or hot* or warm*)).tw,hw.	105364

5	((global* or planet* or earth) adj3 (heating or warming)).tw,hw.	37391
6	(thermal adj3 (expos* or hazard*)).tw,hw.	884
7	(collab* adj4 (govern* or action* or approach* or policy or policies or response*)).tw,hw.	4738
8	((intersection* or inter-section* or intersector* or multi-sector* or inter-sector* or multisector* or cross-sector* or inter-department* or interdepartment* or transdisciplin* or trans-disciplin*) adj4 (approach* or collab* or partner* or action* or response*)).tw,hw.	3781
9	(environ* adj5 (health* or safety)).tw,hw.	58394
10	((public* or region* or local* or city or cities or municipal* or provincial* or province* or territor* or town* or communit*) adj5 (govern* or health* or policy or policies)).tw,hw.	303256
11	(British Columbia or Colombie Britannique or Ontario or Quebec*).mp.	63885
12	((Halifax or Hamilton or London or Victoria or Waterloo or Welland or Whitby or Windsor or Richmond or Milton) not (UK or Britain or United Kingdom or England or Australia or New Zealand)).tw,hw.	13324
13	(Abbotsford or Ajax or Aurora or Barrie or Belleville or Blainville or Brampton or Brantford or Brossard or Burlington or Burnaby or Caledon or Chatham Kent or Chilliwack or Clarington or Coquitlam or Drummondville or Gatineau or Granby or Sudbury or Guelph or Halton Hills or Kamloops or Kawartha Lakes or Kelowna or Kingston or Kitchener or Langley or Laval or Levis or Longueuil or Maple Ridge or Markham or Mirabel or Mississauga or Montreal or New Westminster or Newmarket or Niagara Falls or Norfolk County or North Bay or North Vancouver or Oakville or Oshawa or Ottawa or Peterborough or Pickering or Port Coquitlam or Prince George or Quebec City or Repentigny or Richmond Hill or Saanich or Saguenay or Saint-Hyacinthe or Saint-Jean-sur-Richelieu or Saint-Jerome or Sarnia or Sault Ste Marie or Sherbrooke or St Catharines or Surrey or Terrebonne or Thunder Bay or Toronto or Trois-Rivieres or Vancouver or Vaughan).tw,hw.	21869
14	1 or 2 or 4 or 5	635311
15	7 or 8 or 9 or 10	348625
16	11 or 12 or 13	88685
17	14 and 15 and 16	314
18	**limit 17 to ((english or french) and yr="2005 -Current")**	**294**

Web of Science Core Collection

Entitlements:

- WOS.SCI: 1900 to 2023
- WOS.AHCI: 1975 to 2023
- WOS.BHCI: 2005 to 2023
- WOS.BSCI: 2005 to 2023
- WOS.ESCI: 2005 to 2023
- WOS.ISTP: 1990 to 2023
- WOS.SSCI: 1900 to 2023
- WOS.ISSHP: 1990 to 2023

Searches:

1: ("Arctic amplification" or "Arctic shrinkage" or "carbon footprint" or "carbon offset*" or "carbon sequestration" or "carbon sink" or "carbon sinks" or (chlorofluorocarbon* NEAR/2 (release or concentration* or atmosphere*)) or (climate NEAR/3 (change* or model?ing or predict* or resilience or sensitivity or action* or variability or adaptation or crisis or breakdown)) or deglaciation or desertification or "natural disaster*" or heatwave* or drought or droughts or heat-dome* or (earth NEAR/2 warming) or El Nino or La Nina or "emissions reduc*" or "emissions trading" or flood* or ((glacial or glacier) NEAR/3 (retreat* or melt*)) or "global radiation*" or "global

temperature**" or "global warming" or "greenhouse effect" or "greenhouse gas*" or "heat wave**" or heatwave* or hurricane** or "ice cap melt*" or "ice mass loss*" or "Kyoto Protocol" or "landslide*" or "land slide*" or "mudslide*" or "mud slide*" or high-temp* or (ozone NEAR/2 hole) or "Paris Accord" or "permafrost melt*" or "polar amplification" or "polar ice melt*" or "sea ice shrinkage" or "sea level rise*" or "sea surface warming" or "storm surge*" or "thermohaline circulation" or ((extreme or severe or destructive) NEAR/3 (temperature* or weather* or storm* or hail or wind* or climate or heat or heating or warm* or humidity)) or tornado* or typhoon* or wildfire* or "wild fire*") (Topic) Date Run: Fri Nov 17 2023 11:03:22 GMT-0500 (Eastern Standard Time) Results: 1551694

2: ((increasing or rising or increase* or extreme or "rise in") NEAR/3 (humidity or humid or temperature* or weather* or heat* or hot* or warm*)) (Topic) Date Run: Fri Nov 17 2023 11:03:36 GMT-0500 (Eastern Standard Time) Results: 544526

3: (heat* NEAR/4 (stroke* or hazard* or expos* or wave* or dome or chronic* or prolong* or event* or warn* or shock* or stress* or alert* or alarm*)) (Topic) Date Run: Fri Nov 17 2023 11:03:45 GMT-0500 (Eastern Standard Time) Results: 176481

4: (thermal NEAR/3 (expos* or hazard*)) (Topic) Date Run: Fri Nov 17 2023 11:03:57 GMT-0500 (Eastern Standard Time) Results: 10042

5: heatshock* or hyperthermia* or "heat shock*" or heat-shock (Topic) Date Run: Fri Nov 17 2023 11:04:05 GMT-0500 (Eastern Standard Time) Results: 129198

6: ((global* or planet* or earth) NEAR/3 (heating or warming)) (Topic) Date Run: Fri Nov 17 2023 11:04:14 GMT-0500 (Eastern Standard Time) Results: 78348

7: flood* or hurricane* or polar amplification or sea level rise* or sea surface warming or storm surge or wild fire* or wildfire*. (Topic) Date Run: Fri Nov 17 2023 11:04:51 GMT-0500 (Eastern Standard Time) Results: 300907

8: (collab* NEAR/4 (govern* or action* or approach* or policy or policies or response*)) (Topic) Date Run: Fri Nov 17 2023 11:05:21 GMT-0500 (Eastern Standard Time) Results: 37834

9: ((intersection* or inter-section* or intersector* or multi-sector* or inter-sector* or multisector* or cross-sector* or inter-department* or interdepartment* or transdisciplin* or trans-disciplin*) NEAR/4 (approach* or collab* or partner* or action* or response*)) (Topic) Date Run: Fri Nov 17 2023 11:05:29 GMT-0500 (Eastern Standard Time) Results: 17114

10: (environ* NEAR/5 (health* or safety)) (Topic) Date Run: Fri Nov 17 2023 11:06:03 GMT-0500 (Eastern Standard Time) Results: 154447

11: ((public* or region* or local* or city or cities or municipal* or provincial* or province* or territor* or town* or communit*) NEAR/5 (govern* or health* or policy or policies)) (Topic) Date Run: Fri Nov 17 2023 11:06:12 GMT-0500 (Eastern Standard Time) Results: 880848

12: ((Halifax or Hamilton or London or Victoria or Waterloo or Welland or Whitby or Windsor or Richmond or Milton) NOT (UK or Britain or United Kingdom or England or Australia or New Zealand)) (Topic) Date Run: Fri Nov 17 2023 11:06:31 GMT-0500 (Eastern Standard Time) Results: 175498

13: Abbotsford or Ajax or Aurora or Barrie or Belleville or Blainville or Brampton or Brantford or Brossard or Burlington or Burnaby or Caledon or Chatham Kent or Chilliwack or Clarington or Coquitlam or Drummondville or Gatineau or Granby or Sudbury or Guelph or Halton Hills or Kamloops or Kawartha Lakes or Kelowna or Kingston or Kitchener or Langley or Laval or Levis or Longueuil or Maple Ridge or Markham or Mirabel or Mississauga or

Montreal or New Westminster or Newmarket or Niagara Falls or Norfolk County or North Bay or North Vancouver or Oakville or Oshawa or Ottawa or Peterborough or Pickering or Port Coquitlam or Prince George or Quebec City or Repentigny or Richmond Hill or Saanich or Saguenay or Saint-Hyacinthe or Saint-Jean-sur-Richelieu or Saint-Jerome or Sarnia or Sault Ste Marie or Sherbrooke or St Catharines or Surrey or Terrebonne or Thunder Bay or Toronto or Trois-Rivieres or Vancouver or Vaughan (Topic) Date Run: Fri Nov 17 2023 11:06:45 GMT-0500 (Eastern Standard Time) Results: 150211

14: (British Columbia or Colombie Britannique or Ontario or Quebec*) (Topic) Date Run: Fri Nov 17 2023 11:07:02 GMT-0500 (Eastern Standard Time) Results: 150902

15: #1 OR #2 OR #3 OR #4 OR #5 OR #6 OR #7 Date Run: Fri Nov 17 2023 11:07:52 GMT-0500 (Eastern Standard Time) Results: 2150213

16: #8 OR #9 OR #10 OR #11 Date Run: Fri Nov 17 2023 11:08:07 GMT-0500 (Eastern Standard Time) Results: 1049745

17: #12 OR #13 OR #14 Date Run: Fri Nov 17 2023 11:08:22 GMT-0500 (Eastern Standard Time) Results: 453131

18: #15 AND #16 AND #17 Date Run: Fri Nov 17 2023 11:08:29 GMT-0500 (Eastern Standard Time) Results: 972

19: #15 AND #16 AND #17 and English or French (Languages) Date Run: Fri Nov 17 2023 11:08:52 GMT-0500 (Eastern Standard Time) Results: 966

20: #15 AND #16 AND #17 and English or French (Languages) and 2005 or 2006 or 2007 or 2008 or 2009 or 2010 or 2011 or 2012 or 2013 or 2015 or 2016 or 2017 or 2018 or 2019 or 2020 or 2021 or 2014 or 2022 or 2023 (Publication Years) Date Run: Fri Nov 17 2023 11:09:15 GMT-0500 (Eastern Standard Time) Results: **925**

ProQuest PAIS Index

Search Strategy

Set#: S1
Searched for: noft(Abbotsford or Ajax or Aurora or Barrie or Belleville or Blainville or Brampton or Brantford or Brossard or Burlington or Burnaby or Caledon or Chatham Kent or Chilliwack or Clarington or Coquitlam or Drummondville or Gatineau or Granby or Sudbury or Guelph or Halton Hills or Kamloops or Kawartha Lakes or Kelowna or Kingston or Kitchener or Langley or Laval or Levis or Longueuil or Maple Ridge or Markham or Mirabel or Mississauga or Montreal or New Westminster or Newmarket or Niagara Falls or Norfolk County or North Bay or North Vancouver or Oakville or Oshawa or Ottawa or Peterborough or Pickering or Port Coquitlam or Prince George or Quebec City or Repentigny or Richmond Hill or Saanich or Saguenay or Saint-Hyacinthe or Saint-Jean-sur-Richelieu or Saint-Jerome or Sarnia or Sault Ste Marie or Sherbrooke or St Catharines or Surrey or Terrebonne or Thunder Bay or Toronto or Trois-Rivieres or Vancouver or Vaughan)
Databases: PAIS Index
Results: 24329

Set#: S2
Searched for: noft(((Halifax or Hamilton or London or Victoria or Waterloo or Welland or Whitby or Windsor or Richmond or Milton) not (UK or Britain or United Kingdom or England or Australia or New Zealand)))
Databases: PAIS Index
Results: 26753

Set#: S3
Searched for: noft(British Columbia or Colombie Britannique or Ontario or Quebec*)
Databases: PAIS Index
Results: 16683

Set#: S5
Searched for: noft((environ* NEAR/5 (health* or safety)))
Databases: PAIS Index
Results: 4033

Set#: S6
Searched for: noft(((intersection* or inter-section* or intersector* or multi-sector* or inter-sector* or multisector* or cross-sector* or inter-department* or interdepartment* or transdisciplin* or trans-disciplin*) NEAR/4 (approach* or collab* or partner* or action* or response*)))
Databases: PAIS Index
Results: 1004

Set#: S7
Searched for: noft((collab* NEAR/4 (govern* or action* or approach* or policy or policies or response*)))
Databases: PAIS Index
Results: 134

Set#: S8
Searched for: noft(((global* or planet* or earth) NEAR/3 (heating or warming)))
Databases: PAIS Index
Results: 8093

Set#: S9
Searched for: noft(heatshock* or hyperthermia* or "heat shock*" or heat-shock)
Databases: PAIS Index
Results: 9

Set#: S10
Searched for: noft((thermal NEAR/3 (expos* or hazard*)))
Databases: PAIS Index
Results: 3

Set#: S11
Searched for: noft((heat* NEAR/4 (stroke* or hazard* or expos* or wave* or dome or chronic* or prolong* or event* or warn* or shock* or stress* or alert* or alarm*)))
Databases: PAIS Index
Results: 401

Set#: S12
Searched for: noft(((increasing or rising or increase* or extreme or "rise in") NEAR/3 (humidity or humid or temperature* or weather* or heat* or hot* or warm*)))
Databases: PAIS Index
Results: 1509

Set#: S13
Searched for: noft(("Arctic amplification" or "Arctic shrinkage" or "carbon footprint" or "carbon offset*" or "carbon sequestration" or "carbon sink" or "carbon sinks" or (chlorofluorocarbon* NEAR/2 (release or concentration* or

atmosphere*)) or (climate NEAR/3 (change* or model?ing or predict* or resilience or sensitivity or action* or variability or adaptation or crisis or breakdown)) or deglaciation or desertification or "natural disaster*" or heatwave* or drought or droughts or heat-dome* or (earth N2 warming) or El Nino or La Nina or "emissions reduc*" or "emissions trading" or flood* or ((glacial or glacier) NEAR/3 (retreat* or melt*)) or "global radiation*" or "global temperature**" or "global warming" or "greenhouse effect" or "greenhouse gas*" or "heat wave**" or heatwave* or hurricane** or "ice cap melt*" or "ice mass loss*" or "Kyoto Protocol" or "landslide*" or "land slide*" or "mudslide*" or "mud slide*" or high-temp* or (ozone N2 hole) or "Paris Accord" or "permafrost melt*" or "polar amplification" or "polar ice melt*" or "sea ice shrinkage" or "sea level rise*" or "sea surface warming" or "storm surge*" or "thermohaline circulation" or ((extreme or severe or destructive) NEAR/3 (temperature* or weather* or storm* or hail or wind* or climate or heat or heating or warm* or humidity)) or tornado* or typhoon* or wildfire* or "wild fire*"))
Databases: PAIS Index
Results: 41139

Set#: S14
Searched for: [S1] OR [S2] OR [S3]
Databases: PAIS Index
These databases are searched for part of your query.
Results: 60438

Set#: S15
Searched for: noft(((public* OR region* OR local* OR city OR cities OR municipal* OR provincial* OR province* OR territor* OR town* OR communit*) NEAR/5 (govern* OR health* OR policy OR policies)))
Databases: PAIS Index
Results: 249228

Set#: S16
Searched for: [S5] OR [S6] OR [S7] OR [S8] OR [S15]
Databases: PAIS Index
These databases are searched for part of your query.
Results: 257821

Set#: S17
Searched for: [S8] OR [S9] OR [S10] OR [S11] OR [S12] OR [S13]
Databases: PAIS Index
These databases are searched for part of your query.
Results: 41475

Set#: S18
Searched for: [S14] AND [S16] AND [S17]
Databases: PAIS Index
These databases are searched for part of your query.
Results: 660

Set#: S19
Searched for: ([S14] AND [S16] AND [S17]) AND pd(20050101-20231231)
Databases: PAIS Index
These databases are searched for part of your query.
Results: 594

ProQuest Canadian Research Index

Set#: S1

Searched for: noft(("Arctic amplification" or "Arctic shrinkage" or "carbon footprint" or "carbon offset*" or "carbon sequestration" or "carbon sink" or "carbon sinks" or (chlorofluorocarbon* NEAR/2 (release or concentration* or atmosphere*)) or (climate NEAR/3 (change* or model?ing or predict* or resilience or sensitivity or action* or variability or adaptation or crisis or breakdown)) or deglaciation or desertification or "natural disaster*" or heatwave* or drought or droughts or heat-dome* or (earth N2 warming) or El Nino or La Nina or "emissions reduc*" or "emissions trading" or flood* or ((glacial or glacier) NEAR/3 (retreat* or melt*)) or "global radiation*" or "global temperature**" or "global warming" or "greenhouse effect" or "greenhouse gas*" or "heat wave**" or heatwave* or hurricane** or "ice cap melt*" or "ice mass loss*" or "Kyoto Protocol" or "landslide*" or "land slide*" or "mudslide*" or "mud slide*" or high-temp* or (ozone N2 hole) or "Paris Accord" or "permafrost melt*" or "polar amplification" or "polar ice melt*" or "sea ice shrinkage" or "sea level rise*" or "sea surface warming" or "storm surge*" or "thermohaline circulation" or ((extreme or severe or destructive) NEAR/3 (temperature* or weather* or storm* or hail or wind* or climate or heat or heating or warm* or humidity)) or tornado* or typhoon* or wildfire* or "wild fire*"))
Databases: Canadian Research Index
Results: 3025

Set#: S2
Searched for: noft(((increasing or rising or increase* or extreme or "rise in") NEAR/3 (humidity or humid or temperature* or weather* or heat* or hot* or warm*)))
Databases: Canadian Research Index
Results: 149

Set#: S3
Searched for: noft((heat* NEAR/4 (stroke* or hazard* or expos* or wave* or dome or chronic* or prolong* or event* or warn* or shock* or stress* or alert* or alarm*)))
Databases: Canadian Research Index
Results: 93

Set#: S4
Searched for: noft((thermal NEAR/3 (expos* or hazard*)))
Databases: Canadian Research Index
Results: 6

Set#: S5
Searched for: noft(heatshock* or hyperthermia* or "heat shock*" or heat-shock)
Databases: Canadian Research Index
Results: 8

Set#: S6
Searched for: noft(((global* or planet* or earth) NEAR/3 (heating or warming)))
Databases: Canadian Research Index
Results: 122

Set#: S7
Searched for: noft(flood* or hurricane* or polar amplification or sea level rise* or sea surface warming or storm surge or wild fire* or wildfire*)
Databases: Canadian Research Index
Results: 944

Set#: S8
Searched for: noft((collab* NEAR/4 (govern* or action* or approach* or policy or policies or response*)))
Databases: Canadian Research Index
Results: 6

Set#: S9
Searched for: noft(((intersection* or inter-section* or intersector* or multi-sector* or inter-sector* or multisector* or cross-sector* or inter-department* or interdepartment* or transdisciplin* or trans-disciplin*) NEAR/4 (approach* or collab* or partner* or action* or response*)))
Databases: Canadian Research Index
Results: 52

Set#: S10
Searched for: noft((environ* NEAR/5 (health* or safety)))
Databases: Canadian Research Index
Results: 1367

Set#: S11
Searched for: noft(((public* or region* or local* or city or cities or municipal* or provincial* or province* or territor* or town* or communit*) NEAR/5 (govern* or health* or policy or policies)))
Databases: Canadian Research Index
Results: 20150

Set#: S12
Searched for: noft(((Halifax or Hamilton or London or Victoria or Waterloo or Welland or Whitby or Windsor or Richmond or Milton) NOT (UK or Britain or United Kingdom or England or Australia or New Zealand)))

Databases: Canadian Research Index
Results: 5163

Set#: S13
Searched for: noft(Abbotsford or Ajax or Aurora or Barrie or Belleville or Blainville or Brampton or Brantford or Brossard or Burlington or Burnaby or Caledon or Chatham Kent or Chilliwack or Clarington or Coquitlam or Drummondville or Gatineau or Granby or Sudbury or Guelph or Halton Hills or Kamloops or Kawartha Lakes or Kelowna or Kingston or Kitchener or Langley or Laval or Levis or Longueuil or Maple Ridge or Markham or Mirabel or Mississauga or Montreal or New Westminster or Newmarket or Niagara Falls or Norfolk County or North Bay or North Vancouver or Oakville or Oshawa or Ottawa or Peterborough or Pickering or Port Coquitlam or Prince George or Quebec City or Repentigny or Richmond Hill or Saanich or Saguenay or Saint-Hyacinthe or Saint-Jean-sur-Richelieu or Saint-Jerome or Sarnia or Sault Ste Marie or Sherbrooke or St Catharines or Surrey or Terrebonne or Thunder Bay or
Toronto or Trois-Rivieres or Vancouver or Vaughan)

Databases: Canadian Research Index
Results: 23240

Set#: S14
Searched for: noft((British Columbia or Colombie Britannique or Ontario or Quebec*))
Databases: Canadian Research Index
Results: 100286

Set#: S15
Searched for: [S20] OR [S21] OR [S22] OR [S23] OR [S24] OR [S25] OR [S26]
Databases: Canadian Research Index
    These databases are searched for part of your query.
Results: 3187

Set#: S16

Searched for: [S27] OR [S28] OR [S29] OR [S30]
Databases: Canadian Research Index
These databases are searched for part of your query.
Results: 21200

Set#: S17
Searched for: [S31] OR [S32] OR [S33]
Databases: Canadian Research Index
These databases are searched for part of your query.
Results: 107379

Set#: S18
Searched for: [S34] AND [S35] AND [S36]
Databases: Canadian Research Index
These databases are searched for part of your query.
Results: 100

Set#: S19
Searched for: ([S34] AND [S35] AND [S36]) AND pd(20050101-20231231)
Databases: Canadian Research Index
These databases are searched for part of your query.
Results: 26

ProQuest Policy File

http://myaccess.library.utoronto.ca/login?qurl=https%3A%2F%2Fwww.proquest.com%2Fsearch%2F2502247%3Faccountid%3D14771

Scopus

((TITLE-ABS-KEY ((public* OR region* OR local* OR city OR cities OR municipal* OR provincial* OR province* OR territor* OR town* OR communit*) W/5 (govern* OR health* OR policy OR policies))) OR (TITLE-ABS-KEY (environ* W/5 (health* OR safety))) OR (TITLE-ABS-KEY ((intersection* OR inter-section* OR intersector* OR multi-sector* OR inter-sector* OR multisector* OR cross-sector* OR inter-department* OR interdepartment* OR transdisciplin* OR trans-disciplin*) W/4 (approach* OR collab* OR partner* OR action* OR response*))) OR (TITLE-ABS-KEY (collab* W/4 (govern* OR action* OR approach* OR policy OR policies OR response*)))) AND ((TITLE-ABS-KEY (flood* OR hurricane* OR "polar amplification" OR "sea level rise*" OR "sea surface warming" OR "storm surge" OR "wild fire*" OR wildfire*)) OR (TITLE-ABS-KEY ((global* OR planet* OR earth) W/3 (heating OR warming))) OR (TITLE-ABS-KEY (heatshock* OR hyperthermia* OR "heat shock*" OR heat-shock)) OR (TITLE-ABS-KEY (thermal W/3 (expos* OR hazard*))) OR (TITLE-ABS-KEY ((increasing OR rising OR increase* OR extreme OR "rise in") W/3 (humidity OR humid OR temperature* OR weather* OR heat* OR hot* OR warm*))) OR (TITLE-ABS-KEY ("arctic amplification" OR "arctic shrinkage" OR "carbon footprint" OR "carbon offset*" OR "carbon sequestration" OR "carbon sink" OR "carbon sinks")) OR (TITLE-ABS-KEY (deglaciation OR desertification OR "natural disaster*" OR heatwave* OR drought OR droughts OR heat-dome*)) OR (TITLE-ABS-KEY ("global radiation*" OR "global temperature**" OR "global warming" OR "greenhouse effect" OR "greenhouse gas*" OR "heat wave**" OR heatwave* OR hurricane** OR "ice cap melt*" OR "ice mass loss*" OR "kyoto protocol" OR "landslide*" OR "land slide*" OR "mudslide*" OR "mud slide*" OR high-temp*)) OR (TITLE-ABS-KEY ("el nino" OR "la nina" OR "emissions reduc*" OR "emissions

trading" OR flood*)) OR (TITLE-ABS-KEY ("paris accord" OR "permafrost melt*" OR "polar amplification" OR "polar ice melt*" OR "sea ice shrinkage" OR "sea level rise*" OR "sea surface warming" OR "storm surge*" OR "thermohaline circulation")) OR (TITLE-ABS-KEY (chlorofluorocarbon* W/2 (release OR concentration* OR atmosphere*))) OR (TITLE-ABS-KEY (climate W/3 (change* OR model?ing OR predict* OR resilience OR sensitivity OR action* OR variability OR adaptation OR crisis OR breakdown))) OR (TITLE-ABS-KEY ((glacial OR glacier) W/3 (retreat* OR melt*))) OR (TITLE-ABS-KEY ((extreme OR severe OR destructive) W/3 (temperature* OR weather* OR storm* OR hail OR wind* OR climate OR heat OR heating OR warm* OR humidity))) OR (TITLE-ABS-KEY (ozone W/2 hole*))) AND (((TITLE-ABS-KEY (halifax OR hamilton OR london OR victoria OR waterloo OR welland OR whitby OR windsor OR richmond OR milton) AND NOT TITLE-ABS-KEY (uk OR britain OR united AND kingdom OR england OR australia OR new AND zealand))) OR (TITLE-ABS-KEY (british AND columbia OR colombie AND britannique OR ontario OR quebec*))) AND PUBYEAR > 2004 AND PUBYEAR < 2024 AND (LIMIT-TO (LANGUAGE , "french") OR LIMIT-TO (LANGUAGE , "english"))

Elsevier ENGINEERING Village GEOBASE

Found in Compendex, Inspec, Inspec Archive, GEOBASE, GeoRef, US Patents, EP Patents & WO Patents for 2005-2023: (((((($Abbotsford OR $Ajax OR $Aurora OR $Barrie OR $Belleville OR $Blainville OR $Brampton OR $Brantford OR $Brossard OR $Burlington OR $Burnaby OR $Caledon OR $Chatham $Kent OR $Chilliwack OR $Clarington OR $Coquitlam OR $Drummondville OR $Gatineau OR $Granby OR $Sudbury OR $Guelph OR $Halton $Hills OR $Kamloops OR $Kawartha $Lakes OR $Kelowna OR $Kingston OR $Kitchener OR $Langley OR $Laval OR $Levis OR $Longueuil OR $Maple $Ridge OR $Markham OR $Mirabel OR $Mississauga OR $Montreal OR $New $Westminster OR $Newmarket OR $Niagara $Falls OR $Norfolk $County OR $North $Bay OR $North $Vancouver OR $Oakville OR $Oshawa OR $Ottawa OR $Peterborough OR $Pickering OR $Port $Coquitlam OR $Prince $George OR $Quebec $City OR $Repentigny OR $Saanich OR $Saguenay OR $Saint-Hyacinthe OR $Saint-Jean-sur-Richelieu OR $Saint-Jerome OR $Sarnia OR $Sault $Ste $Marie OR $Sherbrooke OR $St $Catharines OR $Surrey OR $Terrebonne OR $Thunder $Bay OR $Toronto OR $Trois-Rivieres OR $Vancouver OR $Vaughan) WN KY)) OR ((($British $Columbia OR $Colombie $Britannique OR $Ontario OR Quebec*) WN KY)) OR ((((($Halifax OR $Hamilton OR $London OR $Victoria OR $Waterloo OR $Welland OR $Whitby OR $Windsor OR $Richmond OR $Milton) WN KY)) NOT ((($UK OR $Britain OR $United $Kingdom OR $England OR $Australia OR $New $Zealand) WN KY)))))) AND ((((($environmental NEAR/5 $health) WN KY)) OR ((($environment NEAR/5 $health) WN KY)) OR ((($environment NEAR/5 $safety) WN KY)) OR ((($environmental NEAR/5 $safety) WN KY)) OR ((((public* OR region* OR local* OR $city OR $cities OR municipal* OR provincial* OR province* OR territor* OR town* OR communit*) WN KY) AND ((govern* OR health* OR $policy OR $policies) WN KY))) OR ((((intersection* OR inter-section* OR intersector* OR multi-sector* OR inter-sector* OR multisector* OR cross-sector* OR inter-department* OR interdepartment* OR transdisciplin* OR trans-disciplin*) WN KY) AND ((approach* OR collab* OR partner* OR action* OR response*) WN KY))) OR ((((collab*) WN KY) AND ((govern* OR action* OR approach* OR $policy OR $policies OR response*) WN KY))))) AND (((((heatshock* OR hyperthermia* OR {heat shock*} OR $heat-shock) WN KY)) OR ((((($thermal NEAR/3 $hazard) WN KY) OR (($thermal NEAR/3 $hazards) WN KY)) OR (($thermal NEAR/3 $exposure) WN KY))) OR ((((stroke* OR hazard* OR expos* OR wave* OR $dome OR chronic* OR prolong* OR event* OR warn* OR shock* OR stress* OR alert* OR alarm*) WN KY) AND ((heat*) WN KY))) OR (((($increasing OR $rising OR increase* OR $extreme OR {rise in}) WN KY) AND (($humidity OR $humid OR temperature* OR weather* OR heat* OR hot* OR warm*) WN KY))) OR (((flood* OR hurricane* OR $polar $amplification OR $sea $level rise* OR $sea $surface $warming OR $storm $surge OR $wild fire* OR wildfire*) WN KY)) OR ((({Arctic amplification} OR {Arctic shrinkage} OR {carbon footprint} OR {carbon offset*} OR {carbon sequestration} OR {carbon sink} OR {carbon sinks}) WN KY)) OR ((($deglaciation OR $desertification OR {natural disaster*} OR heatwave* OR $drought OR $droughts OR heat-dome*) WN KY)) OR ((({El Nino} OR {La Nina} OR {emissions reduc*} OR {emissions trading} OR flood* OR high-temp*) WN KY)) OR ((({global radiation*} OR {global temperature**} OR {global warming} OR {greenhouse effect} OR {greenhouse gas*} OR {heat wave**} OR heatwave* OR hurricane** OR {ice cap melt*} OR {ice mass loss*} OR {Kyoto Protocol} OR {landslide*} OR {land

slide*} OR {mudslide*} OR {mud slide*}) WN KY)) OR ((({Paris Accord} OR {permafrost melt*} OR {polar amplification} OR {polar ice melt*} OR {sea ice shrinkage} OR {sea level rise*} OR {sea surface warming} OR {storm surge*} OR {thermohaline circulation}) WN KY)) OR ((((chlorofluorocarbon*) WN KY) AND (($release OR concentration* OR atmosphere*) WN KY))) OR (((($climate) WN KY) AND ((change* OR model?ing OR predict* OR $resilience OR $sensitivity OR action* OR $variability OR $adaptation OR $crisis OR $breakdown) WN KY))) OR (((($glacial OR $glacier) WN KY) AND ((retreat* OR melt*) WN KY))) OR ((($ozone NEAR/3 $hole) WN KY)) OR (((($extreme OR $severe OR $destructive) WN KY) AND ((temperature* OR weather* OR storm* OR $hail OR wind* OR $climate OR $heat OR $heating OR warm* OR $humidity) WN KY)))))) + ((geo) WN DB) AND (({english} OR {french}) WN LA)

Results: 1,137 records

Elsevier GEOREF

found in Compendex, Inspec, Inspec Archive, GEOBASE, GeoRef, US Patents, EP Patents & WO Patents for 2005-2023: (((((($Abbotsford OR $Ajax OR $Aurora OR $Barrie OR $Belleville OR $Blainville OR $Brampton OR $Brantford OR $Brossard OR $Burlington OR $Burnaby OR $Caledon OR $Chatham $Kent OR $Chilliwack OR $Clarington OR $Coquitlam OR $Drummondville OR $Gatineau OR $Granby OR $Sudbury OR $Guelph OR $Halton $Hills OR $Kamloops OR $Kawartha $Lakes OR $Kelowna OR $Kingston OR $Kitchener OR $Langley OR $Laval OR $Levis OR $Longueuil OR $Maple $Ridge OR $Markham OR $Mirabel OR $Mississauga OR $Montreal OR $New $Westminster OR $Newmarket OR $Niagara $Falls OR $Norfolk $County OR $North $Bay OR $North $Vancouver OR $Oakville OR $Oshawa OR $Ottawa OR $Peterborough OR $Pickering OR $Port $Coquitlam OR $Prince $George OR $Quebec $City OR $Repentigny OR $Saanich OR $Saguenay OR $Saint-Hyacinthe OR $Saint-Jean-sur-Richelieu OR $Saint-Jerome OR $Sarnia OR $Sault $Ste $Marie OR $Sherbrooke OR $St $Catharines OR $Surrey OR $Terrebonne OR $Thunder $Bay OR $Toronto OR $Trois-Rivieres OR $Vancouver OR $Vaughan) WN KY)) OR ((($British $Columbia OR $Colombie $Britannique OR $Ontario OR Quebec*) WN KY)) OR ((((($Halifax OR $Hamilton OR $London OR $Victoria OR $Waterloo OR $Welland OR $Whitby OR $Windsor OR $Richmond OR $Milton) WN KY)) NOT ((($UK OR $Britain OR $United $Kingdom OR $England OR $Australia OR $New $Zealand) WN KY)))))) AND ((((($environmental NEAR/5 $health) WN KY)) OR ((($environment NEAR/5 $health) WN KY)) OR ((($environment NEAR/5 $safety) WN KY)) OR ((($environmental NEAR/5 $safety) WN KY)) OR ((((public* OR region* OR local* OR $city OR $cities OR municipal* OR provincial* OR province* OR territor* OR town* OR communit*) WN KY) AND ((govern* OR health* OR $policy OR $policies) WN KY))) OR ((((intersection* OR inter-section* OR intersector* OR multi-sector* OR inter-sector* OR multisector* OR cross-sector* OR inter-department* OR interdepartment* OR transdisciplin* OR trans-disciplin*) WN KY) AND ((approach* OR collab* OR partner* OR action* OR response*) WN KY))) OR ((((collab*) WN KY) AND ((govern* OR action* OR approach* OR $policy OR $policies OR response*) WN KY))))) AND (((((heatshock* OR hyperthermia* OR {heat shock*} OR $heat-shock) WN KY)) OR ((((($thermal NEAR/3 $hazard) WN KY) OR (($thermal NEAR/3 $hazards) WN KY)) OR (($thermal NEAR/3 $exposure) WN KY))) OR ((((stroke* OR hazard* OR expos* OR wave* OR $dome OR chronic* OR prolong* OR event* OR warn* OR shock* OR stress* OR alert* OR alarm*) WN KY) AND ((heat*) WN KY))) OR (((($increasing OR $rising OR increase* OR $extreme OR {rise in}) WN KY) AND (($humidity OR $humid OR temperature* OR weather* OR heat* OR hot* OR warm*) WN KY))) OR (((flood* OR hurricane* OR $polar $amplification OR $sea $level rise* OR $sea $surface $warming OR $storm $surge OR $wild fire* OR wildfire*) WN KY)) OR (((({Arctic amplification} OR {Arctic shrinkage} OR {carbon footprint} OR {carbon offset*} OR {carbon sequestration} OR {carbon sink} OR {carbon sinks}) WN KY)) OR ((($deglaciation OR $desertification OR {natural disaster*} OR heatwave* OR $drought OR $droughts OR heat-dome*) WN KY)) OR ((({El Nino} OR {La Nina} OR {emissions reduc*} OR {emissions trading} OR flood* OR high-temp*) WN KY)) OR ((({global radiation*} OR {global temperature**} OR {global warming} OR {greenhouse effect} OR {greenhouse gas*} OR {heat wave**} OR heatwave* OR hurricane** OR {ice cap melt*} OR {ice mass loss*} OR {Kyoto Protocol} OR {landslide*} OR {land slide*} OR {mudslide*} OR {mud slide*}) WN KY)) OR ((({Paris Accord} OR {permafrost melt*} OR {polar amplification} OR {polar ice melt*} OR {sea ice shrinkage} OR {sea level rise*} OR {sea surface warming} OR {storm surge*} OR {thermohaline circulation}) WN KY)) OR ((((chlorofluorocarbon*) WN KY) AND (($release OR concentration* OR atmosphere*) WN KY))) OR (((($climate) WN KY) AND ((change* OR model?ing OR predict* OR $resilience OR $sensitivity OR action* OR $variability OR $adaptation OR $crisis OR $breakdown) WN KY))) OR

(((($glacial OR $glacier) WN KY) AND ((retreat* OR melt*) WN KY))) OR ((($ozone NEAR/3 $hole) WN KY)) OR (((($extreme OR $severe OR $destructive) WN KY) AND ((temperature* OR weather* OR storm* OR $hail OR wind* OR $climate OR $heat OR $heating OR warm* OR $humidity) WN KY)))))) + ((grf) WN DB) AND (({english} OR {french}) WN LA)

Results: 372 records

EBSCO GreenFile

#	Query	Limiters/Expanders	Results
S17	S14 AND S15 AND S16	Limiters - Publication Date: 20050101-20231231<br>Expanders - Apply equivalent subjects<br>Search modes - Boolean/Phrase	**1,318**
S16	S8 OR S9 OR S10 OR S11 OR S12 OR S13	Expanders - Apply equivalent subjects<br>Search modes - Boolean/Phrase	180,942
S15	S4 OR S5 OR S6 OR S7	Expanders - Apply equivalent subjects<br>Search modes - Boolean/Phrase	140,054
S14	S1 OR S2 OR S3	Expanders - Apply equivalent subjects<br>Search modes - Boolean/Phrase	58,475
S13	("Arctic amplification" or "Arctic shrinkage" or "carbon footprint" or "carbon offset*" or "carbon sequestration" or "carbon sink" or "carbon sinks" or (chlorofluorocarbon* N2 (release or concentration* or atmosphere*)) or (climate N3 (change* or model?ing or predict* or resilience or sensitivity or action* or variability or adaptation or crisis or breakdown)) or deglaciation or desertification or "natural disaster*" or heatwave* or drought or droughts or heat-dome* or (earth N2 warming) or El Nino or La Nina or "emissions reduc*" or "emissions trading" or flood* or ((glacial or glacier) N3 (retreat* or melt*)) or "global radiation*" or "global temperature**" or "global warming" or "greenhouse effect" or "greenhouse gas*" or "heat wave**" or heatwave* or hurricane** or "ice cap melt*" or "ice mass loss*" or "Kyoto Protocol" or "landslide*" or "land slide*" or "mudslide*" or "mud slide*" or high-temp* or (ozone N2 hole) or "Paris Accord" or "permafrost melt*" or "polar	Expanders - Apply equivalent subjects<br>Search modes - Boolean/Phrase	170,346

	amplification" or "polar ice melt*" or "sea ice shrinkage" or "sea level rise*" or "sea surface warming" or "storm surge*" or "thermohaline circulation" or ((extreme or severe or destructive) N3 (temperature* or weather* or storm* or hail or wind* or climate or heat or heating or warm* or humidity)) or tornado* or typhoon* or wildfire* or "wild fire*")		
S12	((increasing or rising or increase* or extreme or "rise in") N3 (humidity or humid or temperature* or weather* or heat* or hot* or warm*))	Expanders - Apply equivalent subjects<br>Search modes - Boolean/Phrase	23,615
S11	((global* or planet* or earth) N3 (heating or warming))	Expanders - Apply equivalent subjects<br>Search modes - Boolean/Phrase	19,325
S10	(thermal N3 (expos* or hazard*))	Expanders - Apply equivalent subjects<br>Search modes - Boolean/Phrase	357
S9	(heat* N4 (stroke* or hazard* or expos* or wave* or dome or chronic* or prolong* or event* or warn* or shock* or stress* or alert* or alarm*))	Expanders - Apply equivalent subjects<br>Search modes - Boolean/Phrase	4,395
S8	((global* or planet* or earth) N3 (heating or warming))	Expanders - Apply equivalent subjects<br>Search modes - Boolean/Phrase	19,325
S7	(collab* N4 (govern* or action* or approach* or policy or policies or response*))	Expanders - Apply equivalent subjects<br>Search modes - Boolean/Phrase	1,741
S6	((intersection* or inter-section* or intersector* or multi-sector* or inter-sector* or multisector* or cross-sector* or inter-department* or interdepartment* or transdisciplin* or trans-disciplin*) N4 (approach* or collab* or partner* or action* or response*))	Expanders - Apply equivalent subjects<br>Search modes - Boolean/Phrase	589
S5	(environ* N5 (health* or safety))	Expanders - Apply equivalent subjects<br>Search modes - Boolean/Phrase	96,620
S4	((public* or region* or local* or city or cities or municipal* or provincial* or province* or territor* or town* or communit*) N5 (govern* or health* or policy or policies))	Expanders - Apply equivalent subjects<br>Search modes - Boolean/Phrase	66,972

S3	((Halifax or Hamilton or London or Victoria or Waterloo or Welland or Whitby or Windsor or Richmond or Milton) NOT (UK or Britain or United Kingdom or England or Australia or New Zealand))	Expanders - Apply equivalent subjects<br>Search modes - Boolean/Phrase	16,571
S2	British Columbia or Colombie Britannique or Ontario or Quebec*	Expanders - Apply equivalent subjects<br>Search modes - Boolean/Phrase	29,449
S1	Abbotsford or Ajax or Aurora or Barrie or Belleville or Blainville or Brampton or Brantford or Brossard or Burlington or Burnaby or Caledon or Chatham Kent or Chilliwack or Clarington or Coquitlam or Drummondville or Gatineau or Granby or Sudbury or Guelph or Halton Hills or Kamloops or Kawartha Lakes or Kelowna or Kingston or Kitchener or Langley or Laval or Levis or Longueuil or Maple Ridge or Markham or Mirabel or Mississauga or Montreal or New Westminster or Newmarket or Niagara Falls or Norfolk County or North Bay or North Vancouver or Oakville or Oshawa or Ottawa or Peterborough or Pickering or Port Coquitlam or Prince George or Quebec City or Repentigny or Richmond Hill or Saanich or Saguenay or Saint-Hyacinthe or Saint-Jean-sur-Richelieu or Saint-Jerome or Sarnia or Sault Ste Marie or Sherbrooke or St Catharines or Surrey or Terrebonne or Thunder Bay or Toronto or Trois-Rivieres or Vancouver or Vaughan		

Updated Searches, June 16, 2026

CAB Abstracts

Database(s): **CAB Abstracts** 1973 to 2026 Week 21
Search Strategy:

#	Searches	Results
1	("Arctic amplification" or "Arctic shrinkage" or "carbon footprint" or "carbon offset*" or "carbon sequestration" or "carbon sink" or "carbon sinks" or (chlorofluorocarbon* adj2 (release	816655

	or concentration* or atmosphere*)) or (climate adj3 (change* or model?ing or predict* or resilience or sensitivity or action* or variability or adaptation or crisis or breakdown)) or deglaciation or desertification or "natural disaster*" or heatwave* or drought or droughts or heat-dome* or (earth adj2 warming) or El Nino or La Nina or "emissions reduc*" or "emissions trading" or flood* or ((glacial or glacier) adj3 (retreat* or melt*)) or "global radiation*" or "global temperature**" or "global warming" or "greenhouse effect" or "greenhouse gas*" or "heat wave**" or heatwave* or hurricane** or "ice cap melt*" or "ice mass loss*" or "Kyoto Protocol" or "landslide*" or "land slide*" or "mudslide*" or "mud slide*" or high-temp* or (ozone adj2 hole) or "Paris Accord" or "permafrost melt*" or "polar amplification" or "polar ice melt*" or "sea ice shrinkage" or "sea level rise*" or "sea surface warming" or "storm surge*" or "thermohaline circulation" or ((extreme or severe or destructive) adj3 (temperature* or weather* or storm* or hail or wind* or climate or heat or heating or warm* or humidity)) or tornado* or typhoon* or wildfire* or "wild fire*").mp.	
2	(flood* or hurricane* or polar amplification or sea level rise* or sea surface warming or storm surge or wild fire* or wildfire*).tw,hw.	166427
3	(heat-shock* or hyperthermia* or heatschok* or "heat shock*").tw,hw.	31989
4	((increasing or rising or increase* or extreme or "rise in") adj3 (humidity or humid or temperature* or weather* or heat* or hot* or warm*)).tw,hw.	144423
5	((global* or planet* or earth) adj3 (heating or warming)).tw,hw.	54373

6	(thermal adj3 (expos* or hazard*)).tw,hw.	1371
7	(collab* adj4 (govern* or action* or approach* or policy or policies or response*)).tw,hw.	8711
8	((intersection* or inter-section* or intersector* or multi-sector* or inter-sector* or multisector* or cross-sector* or inter-department* or interdepartment* or transdisciplin* or trans-disciplin*) adj4 (approach* or collab* or partner* or action* or response*)).tw,hw.	7014
9	(environ* adj5 (health* or safety)).tw,hw.	98527
10	((public* or region* or local* or city or cities or municipal* or provincial* or province* or territor* or town* or communit*) adj5 (govern* or health* or policy or policies)).tw,hw.	498396
11	(British Columbia or Colombie Britannique or Ontario or Quebec*).mp.	79613
12	((Halifax or Hamilton or London or Victoria or Waterloo or Welland or Whitby or Windsor or Richmond or Milton) not (UK or Britain or United Kingdom or England or Australia or New Zealand)).tw,hw.	20774
13	(Abbotsford or Ajax or Aurora or Barrie or Belleville or Blainville or Brampton or Brantford or Brossard or Burlington or Burnaby or Caledon or Chatham Kent or Chilliwack or Clarington or Coquitlam or Drummondville or Gatineau or Granby or Sudbury or Guelph or Halton Hills or Kamloops or Kawartha Lakes or Kelowna or Kingston or Kitchener or Langley or Laval or Levis or Longueuil or Maple Ridge or Markham or Mirabel or Mississauga or Montreal or New Westminster or Newmarket or Niagara Falls or Norfolk County or North Bay or North Vancouver or Oakville or Oshawa or Ottawa or Peterborough or Pickering or Port Coquitlam or Prince George or	30630

	Quebec City or Repentigny or Richmond Hill or Saanich or Saguenay or Saint-Hyacinthe or Saint-Jean-sur-Richelieu or Saint-Jerome or Sarnia or Sault Ste Marie or Sherbrooke or St Catharines or Surrey or Terrebonne or Thunder Bay or Toronto or Trois-Rivieres or Vancouver or Vaughan).tw,hw.	
14	1 or 2 or 4 or 5	895955
15	7 or 8 or 9 or 10	574266
16	11 or 12 or 13	118245
17	14 and 15 and 16	500
18	limit 17 to ((english or french) and yr="2005 -Current")	471

MEDLINE

Database(s): **Ovid MEDLINE(R) ALL** 1946 to June 15, 2026
Search Strategy:

#	Searches	Results
1	carbon cycle/ or carbon sequestration/ or climate change/ or global warming/ or droughts/ or El Nino-Southern Oscillation/ or floods/ or greenhouse effect/ or Hot Temperature/ or ("Arctic amplification" or "Arctic shrinkage" or "carbon footprint" or "carbon offset*" or "carbon sequestration" or "carbon sink" or "carbon sinks" or (chlorofluorocarbon* adj2 (release or concentration* or atmosphere*)) or (climate adj3 (change* or model?ing or predict* or resilience or sensitivity or action* or variability or adaptation or crisis or breakdown)) or deglaciation or desertification or "natural disaster*" or heatwave* or drought or droughts or heat-dome* or (earth adj2 warming) or El Nino or La Nina or "emissions reduc*" or "emissions trading" or flood* or high-temp* or ((glacial or glacier) adj3 (retreat* or melt*)) or "global radiation*" or "global temperature**" or "global warming" or "greenhouse effect" or	453259

	"greenhouse gas*" or "heat wave**" or heatwave* or hurricane** or "ice cap melt*" or "ice mass loss*" or "Kyoto Protocol" or "landslide*" or "land slide*" or "mudslide*" or "mud slide*" or (ozone adj2 hole) or "Paris Accord" or "permafrost melt*" or "polar amplification" or "polar ice melt*" or "sea ice shrinkage" or "sea level rise*" or "sea surface warming" or "storm surge*" or "thermohaline circulation" or ((extreme or severe or destructive) adj3 (temperature* or weather* or storm* or hail or wind* or climate or heat or heating or warm* or humidity)) or tornado* or typhoon* or wildfire* or "wild fire*").mp.	
2	(flood* or hurricane* or polar amplification or sea level rise* or sea surface warming or storm surge or wild fire* or wildfire*).tw,kf.	41987
3	((increasing or rising or increase* or extreme or "rise in") adj3 (humidity or humid or temperature* or weather* or heat* or hot* or warm*)).tw,kf.	111936
4	(heat* adj4 (stroke* or hazard* or expos* or wave* or dome or chronic* or prolong* or event* or warn* or shock* or stress* or alert* or alarm*)).tw,kf.	101675
5	(thermal adj3 (expos* or hazard*)).tw,kf.	2645
6	(heatshock* or hyperthermia* or "heat shock*" or heat-shock).tw,kf.	94145
7	((global* or planet* or earth) adj3 (heating or warming)).tw,kf.	19710
8	Extreme Heat/	1133
9	Hot Temperature/	135947
10	Temperature/	282829
11	Climate/	28038
12	limit 11 to yr="1993 - 2009"	7207
13	Heat-Shock Response/	11849
14	public health/ or public health practice/	109214

15	public policy/ or environmental policy/ or health policy/ or policy making/	126121
16	(collab* adj4 (govern* or action* or approach* or policy or policies or response*)).tw,kf.	16959
17	government/ or government agencies/ or local government/ or state government/	45923
18	((intersection* or inter-section* or intersector* or multi-sector* or inter-sector* or multisector* or cross-sector* or inter-department* or interdepartment* or transdisciplin* or trans-disciplin*) adj4 (approach* or collab* or partner* or action* or response*)).tw,kf.	10700
19	(environ* adj5 (health* or safety)).tw,kf.	114921
20	((public* or region* or local* or city or cities or municipal* or provincial* or province* or territor* or town* or communit*) adj5 (govern* or health* or policy or policies)).tw,kf.	769004
21	british columbia/ or ontario/ or quebec/	63501
22	(British Columbia or Colombie Britannique or Ontario or Quebec*).mp.	98669
23	(Abbotsford or Ajax or Aurora or Barrie or Belleville or Blainville or Brampton or Brantford or Brossard or Burlington or Burnaby or Caledon or Chatham Kent or Chilliwack or Clarington or Coquitlam or Drummondville or Gatineau or Granby or Sudbury or Guelph or Halton Hills or Kamloops or Kawartha Lakes or Kelowna or Kingston or Kitchener or Langley or Laval or Levis or Longueuil or Maple Ridge or Markham or Mirabel or Mississauga or Montreal or New Westminster or Newmarket or Niagara Falls or Norfolk County or North Bay or North Vancouver or Oakville or Oshawa or Ottawa or Peterborough or Pickering or Port Coquitlam or Prince George or Quebec City or Repentigny or	81987

	Saanich or Saguenay or Saint-Hyacinthe or Saint-Jean-sur-Richelieu or Saint-Jerome or Sarnia or Sault Ste Marie or Sherbrooke or St Catharines or Surrey or Terrebonne or Thunder Bay or Toronto or Trois-Rivieres or Vancouver or Vaughan).tw,kw.	
24	((Halifax or Hamilton or London or Victoria or Waterloo or Welland or Whitby or Windsor or Richmond or Milton) not (UK or Britain or United Kingdom or England or Australia or New Zealand)).tw,kw.	64105
25	1 or 2 or 3 or 4 or 5 or 6 or 7 or 8 or 9 or 10 or 12 or 13	848825
26	14 or 15 or 16 or 17 or 18 or 19 or 20	1029311
27	21 or 22 or 23 or 24	226570
28	25 and 26 and 27	433
29	limit 28 to yr="2005 - 2026"	417
30	limit 29 to (english or french)	415

Web of Science Core Collection

Web of Science Search Strategy (v0.1)

Database: Web of Science Core Collection

Entitlements:

- WOS.IC: 1993 to 2026
- WOS.CCR: 1985 to 2026
- WOS.SCI: 1900 to 2026
- WOS.AHCI: 1975 to 2026
- WOS.BHCI: 2005 to 2026
- WOS.BSCI: 2005 to 2026
- WOS.ESCI: 2005 to 2026
- WOS.ISTP: 1990 to 2026
- WOS.SSCI: 1900 to 2026
- WOS.ISSHP: 1990 to 2026

Searches:
Search: #12 AND #13 AND #14 and 2005 or 2006 or 2007 or 2008 or 2009 or 2010 or 2011 or 2012 or 2013 or 2014 or 2015 or 2016 or 2017 or 2018 or 2019 or 2020 or 2021 or 2022 or 2023 or 2024 or 2025 or 2026 (Publication Years) Date Run: Tue Jun 16 2026 11:17:36 GMT-0400 (Eastern Daylight Time) Results: 1358
Search: #12 AND #13 AND #14 Date Run: Tue Jun 16 2026 11:16:23 GMT-0400 (Eastern Daylight Time) Results: 1405

Search: #12 AND #13 AND #14 Date Run: Tue Jun 16 2026 11:16:17 GMT-0400 (Eastern Daylight Time) Results: 1405
Search: ((Halifax or Hamilton or London or Victoria or Waterloo or Welland or Whitby or Windsor or Richmond or Milton) NOT (UK or Britain or United Kingdom or England or Australia or New Zealand)) (Topic) OR Abbotsford or Ajax or Aurora or Barrie or Belleville or Blainville or Brampton or Brantford or Brossard or Burlington or Burnaby or Caledon or Chatham Kent or Chilliwack or Clarington or Coquitlam or Drummondville or Gatineau or Granby or Sudbury or Guelph or Halton Hills or Kamloops or Kawartha Lakes or Kelowna or Kingston or Kitchener or Langley or Laval or Levis or Longueuil or Maple Ridge or Markham or Mirabel or Mississauga or Montreal or New Westminster or Newmarket or Niagara Falls or Norfolk County or North Bay or North Vancouver or Oakville or Oshawa or Ottawa or Peterborough or Pickering or Port Coquitlam or Prince George or Quebec City or Repentigny or Richmond Hill or Saanich or Saguenay or Saint-Hyacinthe or Saint-Jean-sur-Richelieu or Saint-Jerome or Sarnia or Sault Ste Marie or Sherbrooke or St Catharines or Surrey or Terrebonne or Thunder Bay or Toronto or Trois-Rivieres or Vancouver or Vaughan (Topic) OR (British Columbia or Colombie Britannique or Ontario or Quebec*) (Topic) Date Run: Tue Jun 16 2026 11:15:18 GMT-0400 (Eastern Daylight Time) Results: 538414
Search: (collab* NEAR/4 (govern* or action* or approach* or policy or policies or response*)) (Topic) OR ((intersection* or inter-section* or intersector* or multi-sector* or inter-sector* or multisector* or cross-sector* or inter-department* or interdepartment* or transdisciplin* or trans-disciplin*) NEAR/4 (approach* or collab* or partner* or action* or response*)) (Topic) OR (environ* NEAR/5 (health* or safety)) (Topic) OR ((public* or region* or local* or city or cities or municipal* or provincial* or province* or territor* or town* or communit*) NEAR/5 (govern* or health* or policy or policies)) (Topic) Date Run: Tue Jun 16 2026 11:11:02 GMT-0400 (Eastern Daylight Time) Results: 1419340
Search: ("Arctic amplification" or "Arctic shrinkage" or "carbon footprint" or "carbon offset*" or "carbon sequestration" or "carbon sink" or "carbon sinks" or (chlorofluorocarbon* NEAR/2 (release or concentration* or atmosphere*)) or (climate NEAR/3 (change* or model?ing or predict* or resilience or sensitivity or action* or variability or adaptation or crisis or breakdown)) or deglaciation or desertification or "natural disaster*" or heatwave* or drought or droughts or heat-dome* or (earth NEAR/2 warming) or El Nino or La Nina or "emissions reduc*" or "emissions trading" or flood* or ((glacial or glacier) NEAR/3 (retreat* or melt*)) or "global radiation*" or "global temperature**" or "global warming" or "greenhouse effect" or "greenhouse gas*" or "heat wave**" or heatwave* or hurricane** or "ice cap melt*" or "ice mass loss*" or "Kyoto Protocol" or "landslide*" or "land slide*" or "mudslide*" or "mud slide*" or high-temp* or (ozone NEAR/2 hole) or "Paris Accord" or "permafrost melt*" or "polar amplification" or "polar ice melt*" or "sea ice shrinkage" or "sea level rise*" or "sea surface warming" or "storm surge*" or "thermohaline circulation" or ((extreme or severe or destructive) NEAR/3 (temperature* or weather* or storm* or hail or wind* or climate or heat or heating or warm* or humidity)) or tornado* or typhoon* or wildfire* or "wild fire*") (Topic) OR ((increasing or rising or increase* or extreme or "rise in") NEAR/3 (humidity or humid or temperature* or weather* or heat* or hot* or warm*)) (Topic) OR (heat* NEAR/4 (stroke* or hazard* or expos* or wave* or dome or chronic* or prolong* or event* or warn* or shock* or stress* or alert* or alarm*)) (Topic) OR (thermal NEAR/3 (expos* or hazard*)) (Topic) OR heatshock* or hyperthermia* or "heat shock*" or heat-shock* (Topic) OR ((global* or planet* or earth) NEAR/3 (heating or warming)) (Topic) OR flood* or hurricane* or polar amplification or sea level rise* or sea surface warming or storm surge or wild fire* or wildfire* (Topic) Date Run: Tue Jun 16 2026 11:08:13 GMT-0400 (Eastern Daylight Time) Results: 2743709
Search: #8 AND #2 AND #1 Date Run: Tue Jun 16 2026 10:25:49 GMT-0400 (Eastern Daylight Time) Results: 498
Search: #8 AND #2 AND #1 Date Run: Tue Jun 16 2026 10:23:12 GMT-0400 (Eastern Daylight Time) Results: 498
Search: #8 AND #2 AND #1 Date Run: Tue Jun 16 2026 10:23:04 GMT-0400 (Eastern Daylight Time) Results: 498
Search: Abbotsford or Ajax or Aurora or Barrie or Belleville or Blainville or Brampton or bransford or Brossard or Burlington or Burnaby or calydon or Chatham Kent or Chilliwack or carington or Coquitlam or drummondvill or Gatineau or granny or Sudbury or Guelph or Halton Hills or Kamloops or kawardha Lakes or Kelowna or Kingston or Kitchener or Langley or Laval or Levis or longueiul or Maple Ridge or Markham or Mirabel or Mississauga or Montreal or New Westminster or Newmarket or Niagara Falls or Norfolk County or North Bay or North Vancouver or oroville or ohsawa or Ottawa or Peterborough or Pickering or Port Coquitlam or Prince George or Quebec City or repenting or Richmond Hill or Saanich or Saguenay or Saint-Hyacinthe or Saint-Jean-sur-Richelieu or Saint-Jerome or Sarnia or Sault Ste Marie or Sherbrooke or St catherines or Surrey or Terrebonne or Thunder Bay or Toronto or Trois-Rivieres or Vancouver or Vaughan (Topic) OR British Columbia or Colombie Britannique or Ontario or

Quebec* (Topic) OR ((Halifax or Hamilton or London or Victoria or Waterloo or Welland or Whitby or Windsor or Richmond or Milton) NOT (UK or Britain or United Kingdom or England or Australia or New Zealand)) (Topic) Date Run: Tue Jun 16 2026 10:22:25 GMT-0400 (Eastern Daylight Time) Results: 541449

Search: Abbotsford or Ajax or Aurora or Barrie or Belleville or Blainville or Brampton or Brantford or Brossard or Burlington or Burnaby or Caledon or Chatham Kent or Chilliwack or Clarington or Coquitlam or Drummondville or Gatineau or Granby or Sudbury or Guelph or Halton Hills or Kamloops or Kawartha Lakes or Kelowna or Kingston or Kitchener or Langley or Laval or Levis or Longueuil or Maple Ridge or Markham or Mirabel or Mississauga or Montreal or New Westminster or Newmarket or Niagara Falls or Norfolk County or North Bay or North Vancouver or Oakville or Oshawa or Ottawa or Peterborough or Pickering or Port Coquitlam or Prince George or Quebec City or Repentigny or Richmond Hill or Saanich or Saguenay or Saint-Hyacinthe or Saint-Jean-sur-Richelieu or Saint-Jerome or Sarnia or Sault Ste Marie or Sherbrooke or St Catharines or Surrey or Terrebonne or Thunder Bay or Toronto or Trois-Rivieres or Vancouver or Vaughan (Topic) OR British Columbia or Colombie Britannique or Ontario or Quebec* (Topic) OR ((Halifax or Hamilton or London or Victoria or Waterloo or Welland or Whitby or Windsor or Richmond or Milton) NOT (UK or Britain or United Kingdom or England or Australia or New Zealand)) (Topic) Date Run: Tue Jun 16 2026 10:22:19 GMT-0400 (Eastern Daylight Time) Results: 538414

Search: #3 AND #2 AND #1 Date Run: Tue Jun 16 2026 10:22:03 GMT-0400 (Eastern Daylight Time) Results: 403

Search: #3 AND #2 AND #1 Date Run: Tue Jun 16 2026 10:21:27 GMT-0400 (Eastern Daylight Time) Results: 403

Search: #3 AND #2 AND #1 Date Run: Tue Jun 16 2026 10:21:22 GMT-0400 (Eastern Daylight Time) Results: 403

Search: Abbotsford or Ajax or Aurora or Barrie or Belleville or Blainville or Brampton or Brantford or Brossard or Burlington or Burnaby or Caledon or Chatham Kent or Chilliwack or Clarington or Coquitlam or Drummondville or Gatineau or Granby or Sudbury or Guelph or Halton Hills or Kamloops or Kawartha Lakes or Kelowna or Kingston or Kitchener or Langley or Laval or Levis or Longueuil or Maple Ridge or Markham or Mirabel or Mississauga or Montreal or New Westminster or Newmarket or Niagara Falls or Norfolk County or North Bay or North Vancouver or Oakville or Oshawa or Ottawa or Peterborough or Pickering or Port Coquitlam or Prince George or Quebec City or Repentigny or Richmond Hill or Saanich or Saguenay or Saint-Hyacinthe or Saint-Jean-sur-Richelieu or Saint-Jerome or Sarnia or Sault Ste Marie or Sherbrooke or St Catharines or Surrey or Terrebonne or Thunder Bay or Toronto or Trois-Rivieres or Vancouver or Vaughan (Topic) OR British Columbia or Colombie Britannique or Ontario or Quebec* (Topic) Date Run: Tue Jun 16 2026 10:20:52 GMT-0400 (Eastern Daylight Time) Results: 342055

Search: (collab* NEAR/4 (govern* or action* or approach* or policy or policies or response*)) (Topic) OR ((intersection* or inter-section* or intersector* or multi-sector* or inter-sector* or multisector* or cross-sector* or inter-department* or interdepartment* or transdisciplin* or trans-disciplin*) NEAR/4 (approach* or collab* or partner* or action* or response*)) (Topic) OR (environ* NEAR/5 (health* or safety)) (Topic) OR ((public* or region* or local* or city or cities or municipal* or provincial* or province* or territor* or town* or communit*) NEAR/5 (govern* or health* or policy or policies)) (Topic) Date Run: Tue Jun 16 2026 10:20:19 GMT-0400 (Eastern Daylight Time) Results: 1419340

Search: TS=(("Arctic amplification" or "Arctic shrinkage" or "carbon footprint" or "carbon offset*" or "carbon sequestration" or "carbon sink" or "carbon sinks" or (chlorofluorocarbon* NEAR/2 (release or concentration* or atmosphere*)) or (climate NEAR/3 (change* or model?ing or predict* or resilience or sensitivity or action* or variability or adaptation or crisis or breakdown)) or deglaciation or desertification or "natural disaster*" or heatwave* or drought or droughts or heat-dome* or (earth NEAR/2 warming) or El Nino or La Nina or "emissions reduc*" or "emissions trading" or flood* or ((glacial or glacier) NEAR/3 (retreat* or melt*)) or "global radiation*" or "global temperature**" or "global warming" or "greenhouse effect" or "greenhouse gas*" or "heat wave**" or heatwave* or hurricane** or "ice cap melt*" or "ice mass loss*" or "Kyoto Protocol" or "landslide*" or "land slide*" or "mudslide*" or "mud slide*" or high-temp* or (ozone NEAR/2 hole) or "Paris Accord" or "permafrost melt*" or "polar amplification" or "polar ice melt*" or "sea ice shrinkage" or "sea level rise*" or "sea surface warming" or "storm surge*" or "thermohaline circulation" or ((extreme or severe or destructive) NEAR/3 (temperature* or weather* or storm* or hail or wind* or climate or heat or heating or warm* or humidity)) or tornado* or typhoon* or wildfire* or "wild fire*") (Topic)) OR TS=(((increasing or rising or increase* or extreme or "rise in") NEAR/3 (humidity or humid or temperature* or weather* or heat* or hot* or warm*)) (Topic)) OR TS=((heat* NEAR/4 (stroke* or hazard* or expos* or wave* or dome or chronic* or prolong* or event* or warn* or shock* or stress* or alert* or alarm*))) OR TS=((thermal NEAR/3 (expos* or hazard*))) OR TS=(heatshock* or hyperthermia* or "heat shock*" or heat-shock) OR TS=(((global* or planet* or earth) NEAR/3 (heating or warming))) OR TS=(flood* or hurricane* or polar amplification or sea level rise* or sea surface warming or storm

surge or wild fire* or wildfire*) Date Run: Tue Jun 16 2026 10:17:54 GMT-0400 (Eastern Daylight Time) Results: 754336

ProQuest Canadian Research Index

ProQUEST Pollicy File Index

(noft(("Arctic amplification" OR "Arctic shrinkage" OR "carbon footprint" OR "carbon offset*" OR "carbon sequestration" OR "carbon sink" OR "carbon sinks" OR (chlorofluorocarbon* NEAR/2 (release OR concentration* OR atmosphere*)) OR (climate NEAR/3 (change* OR model?ing OR predict* OR resilience OR sensitivity OR action* OR variability OR adaptation OR crisis OR breakdown)) OR deglaciation OR desertification OR "natural disaster*" OR heatwave* OR drought OR droughts OR heat-dome* OR (earth N2 warming) OR El Nino OR La Nina OR "emissions reduc*" OR "emissions trading" OR flood* OR ((glacial OR glacier) NEAR/3 (retreat* OR melt*)) OR "global radiation*" OR "global temperature**" OR "global warming" OR "greenhouse effect" OR "greenhouse gas*" OR "heat wave**" OR heatwave* OR hurricane** OR "ice cap melt*" OR "ice mass loss*" OR "Kyoto Protocol" OR "landslide*" OR "land slide*" OR "mudslide*" OR "mud slide*" OR high-temp* OR (ozone N2 hole) OR "Paris Accord" OR "permafrost melt*" OR "polar amplification" OR "polar ice melt*" OR "sea ice shrinkage" OR "sea level rise*" OR "sea surface warming" OR "storm surge*" OR "thermohaline circulation" OR ((extreme OR severe OR destructive) NEAR/3 (temperature* OR weather* OR storm* OR hail OR wind* OR climate OR heat OR heating OR warm* OR humidity)) OR tornado* OR typhoon* OR wildfire* OR "wild fire*")) OR noft(((increasing OR rising OR increase* OR extreme OR "rise in") NEAR/3 (humidity OR humid OR temperature* OR weather* OR heat* OR hot* OR warm*))) OR noft((heat* NEAR/4 (stroke* OR hazard* OR expos* OR wave* OR dome OR chronic* OR prolong* OR event* OR warn* OR shock* OR stress* OR alert* OR alarm*))) OR noft((thermal NEAR/3 (expos* OR hazard*))) OR noft(heatshock* OR hyperthermia* OR "heat shock*" OR heat-shock) OR noft(((global* OR planet* OR earth) NEAR/3 (heating OR warming))) OR noft(flood* OR hurricane* OR polar amplification OR sea level rise* OR sea surface warming OR storm surge OR wild fire* OR wildfire*.)) AND (noft((collab* NEAR/4 (govern* OR action* OR approach* OR policy OR policies OR response*))) OR noft(((intersection* OR inter-section* OR intersector* OR multi-sector* OR inter-sector* OR multisector* OR cross-sector* OR inter-department* OR interdepartment* OR transdisciplin* OR trans-disciplin*) NEAR/4 (approach* OR collab* OR partner* OR action* OR response*))) OR noft((environ* NEAR/5 (health* OR safety))) OR noft(((public* OR region* OR local* OR city OR cities OR municipal* OR provincial* OR province* OR territor* OR town* OR communit*) NEAR/5 (govern* OR health* OR policy OR policies)))) AND (noft(((Halifax OR Hamilton OR London OR Victoria OR Waterloo OR Welland OR Whitby OR Windsor OR Richmond OR Milton) NOT (UK OR Britain OR United Kingdom OR England OR Australia OR New Zealand))) OR noft(Abbotsford OR Ajax OR Aurora OR Barrie OR Belleville OR Blainville OR Brampton OR Brantford OR Brossard OR Burlington OR Burnaby OR Caledon OR Chatham Kent OR Chilliwack OR Clarington OR Coquitlam OR Drummondville OR Gatineau OR Granby OR Sudbury OR Guelph OR Halton Hills OR Kamloops OR Kawartha Lakes OR Kelowna OR Kingston OR Kitchener OR Langley OR Laval OR Levis OR Longueuil OR Maple Ridge OR Markham OR Mirabel OR Mississauga OR Montreal OR New Westminster OR Newmarket OR Niagara Falls OR Norfolk County OR North Bay OR North Vancouver OR Oakville OR Oshawa OR Ottawa OR Peterborough OR Pickering OR Port Coquitlam OR Prince George OR Quebec City OR Repentigny OR Richmond Hill OR Saanich OR Saguenay OR Saint-Hyacinthe OR Saint-Jean-sur-Richelieu OR Saint-Jerome OR Sarnia OR Sault Ste Marie OR Sherbrooke OR St Catharines OR Surrey OR Terrebonne OR Thunder Bay OR Toronto OR Trois-Rivieres OR Vancouver OR Vaughan) OR noft((British Columbia OR Colombie Britannique OR Ontario OR Quebec*)))
22 results, 2006-2026

Proquest PAIS Index

(noft(("Arctic amplification" OR "Arctic shrinkage" OR "carbon footprint" OR "carbon offset*" OR "carbon sequestration" OR "carbon sink" OR "carbon sinks" OR (chlorofluorocarbon* NEAR/2 (release OR concentration* OR atmosphere*)) OR (climate NEAR/3 (change* OR model?ing OR predict* OR resilience OR sensitivity OR action* OR variability OR adaptation OR crisis OR breakdown)) OR deglaciation OR desertification OR "natural disaster*" OR heatwave* OR drought OR droughts OR heat-dome* OR (earth N2 warming) OR El Nino OR La Nina OR

"emissions reduc*" OR "emissions trading" OR flood* OR ((glacial OR glacier) NEAR/3 (retreat* OR melt*)) OR "global radiation*" OR "global temperature**" OR "global warming" OR "greenhouse effect" OR "greenhouse gas*" OR "heat wave**" OR heatwave* OR hurricane** OR "ice cap melt*" OR "ice mass loss*" OR "Kyoto Protocol" OR "landslide*" OR "land slide*" OR "mudslide*" OR "mud slide*" OR high-temp* OR (ozone N2 hole) OR "Paris Accord" OR "permafrost melt*" OR "polar amplification" OR "polar ice melt*" OR "sea ice shrinkage" OR "sea level rise*" OR "sea surface warming" OR "storm surge*" OR "thermohaline circulation" OR ((extreme OR severe OR destructive) NEAR/3 (temperature* OR weather* OR storm* OR hail OR wind* OR climate OR heat OR heating OR warm* OR humidity)) OR tornado* OR typhoon* OR wildfire* OR "wild fire*")) OR noft(((increasing OR rising OR increase* OR extreme OR "rise in") NEAR/3 (humidity OR humid OR temperature* OR weather* OR heat* OR hot* OR warm*))) OR noft((heat* NEAR/4 (stroke* OR hazard* OR expos* OR wave* OR dome OR chronic* OR prolong* OR event* OR warn* OR shock* OR stress* OR alert* OR alarm*))) OR noft((thermal NEAR/3 (expos* OR hazard*))) OR noft(heatshock* OR hyperthermia* OR "heat shock*" OR heat-shock) OR noft(((global* OR planet* OR earth) NEAR/3 (heating OR warming))) OR noft(flood* OR hurricane* OR polar amplification OR sea level rise* OR sea surface warming OR storm surge OR wild fire* OR wildfire*.)) AND (noft((collab* NEAR/4 (govern* OR action* OR approach* OR policy OR policies OR response*))) OR noft(((intersection* OR inter-section* OR intersector* OR multi-sector* OR inter-sector* OR multisector* OR cross-sector* OR inter-department* OR interdepartment* OR transdisciplin* OR trans-disciplin*) NEAR/4 (approach* OR collab* OR partner* OR action* OR response*))) OR noft((environ* NEAR/5 (health* OR safety))) OR noft(((public* OR region* OR local* OR city OR cities OR municipal* OR provincial* OR province* OR territor* OR town* OR communit*) NEAR/5 (govern* OR health* OR policy OR policies)))) AND (noft(((Halifax OR Hamilton OR London OR Victoria OR Waterloo OR Welland OR Whitby OR Windsor OR Richmond OR Milton) NOT (UK OR Britain OR United Kingdom OR England OR Australia OR New Zealand))) OR noft(Abbotsford OR Ajax OR Aurora OR Barrie OR Belleville OR Blainville OR Brampton OR Brantford OR Brossard OR Burlington OR Burnaby OR Caledon OR Chatham Kent OR Chilliwack OR Clarington OR Coquitlam OR Drummondville OR Gatineau OR Granby OR Sudbury OR Guelph OR Halton Hills OR Kamloops OR Kawartha Lakes OR Kelowna OR Kingston OR Kitchener OR Langley OR Laval OR Levis OR Longueuil OR Maple Ridge OR Markham OR Mirabel OR Mississauga OR Montreal OR New Westminster OR Newmarket OR Niagara Falls OR Norfolk County OR North Bay OR North Vancouver OR Oakville OR Oshawa OR Ottawa OR Peterborough OR Pickering OR Port Coquitlam OR Prince George OR Quebec City OR Repentigny OR Richmond Hill OR Saanich OR Saguenay OR Saint-Hyacinthe OR Saint-Jean-sur-Richelieu OR Saint-Jerome OR Sarnia OR Sault Ste Marie OR Sherbrooke OR St Catharines OR Surrey OR Terrebonne OR Thunder Bay OR Toronto OR Trois-Rivieres OR Vancouver OR Vaughan) OR noft((British Columbia OR Colombie Britannique OR Ontario OR Quebec*)))

Results 704
Dates 2005-2026

Proquest Canadian Research Index

(noft(("Arctic amplification" OR "Arctic shrinkage" OR "carbon footprint" OR "carbon offset*" OR "carbon sequestration" OR "carbon sink" OR "carbon sinks" OR (chlorofluorocarbon* NEAR/2 (release OR concentration* OR atmosphere*)) OR (climate NEAR/3 (change* OR model?ing OR predict* OR resilience OR sensitivity OR action* OR variability OR adaptation OR crisis OR breakdown)) OR deglaciation OR desertification OR "natural disaster*" OR heatwave* OR drought OR droughts OR heat-dome* OR (earth N2 warming) OR El Nino OR La Nina OR "emissions reduc*" OR "emissions trading" OR flood* OR ((glacial OR glacier) NEAR/3 (retreat* OR melt*)) OR "global radiation*" OR "global temperature**" OR "global warming" OR "greenhouse effect" OR "greenhouse gas*" OR "heat wave**" OR heatwave* OR hurricane** OR "ice cap melt*" OR "ice mass loss*" OR "Kyoto Protocol" OR "landslide*" OR "land slide*" OR "mudslide*" OR "mud slide*" OR high-temp* OR (ozone N2 hole) OR "Paris Accord" OR "permafrost melt*" OR "polar amplification" OR "polar ice melt*" OR "sea ice shrinkage" OR "sea level rise*" OR "sea surface warming" OR "storm surge*" OR "thermohaline circulation" OR ((extreme OR severe OR destructive) NEAR/3 (temperature* OR weather* OR storm* OR hail OR wind* OR climate OR heat OR heating OR warm* OR humidity)) OR tornado* OR typhoon* OR wildfire* OR "wild fire*")) OR noft(((increasing OR rising OR increase* OR extreme OR "rise in") NEAR/3 (humidity OR humid OR temperature* OR weather* OR heat* OR hot* OR warm*))) OR noft((heat* NEAR/4 (stroke* OR hazard* OR expos* OR wave* OR dome OR chronic* OR prolong* OR event* OR warn* OR shock* OR stress* OR alert* OR alarm*))) OR noft((thermal NEAR/3 (expos* OR

hazard*))) OR noft(heatshock* OR hyperthermia* OR "heat shock*" OR heat-shock) OR noft(((global* OR planet* OR earth) NEAR/3 (heating OR warming))) OR noft(flood* OR hurricane* OR polar amplification OR sea level rise* OR sea surface warming OR storm surge OR wild fire* OR wildfire*.)) AND (noft((collab* NEAR/4 (govern* OR action* OR approach* OR policy OR policies OR response*))) OR noft(((intersection* OR inter-section* OR intersector* OR multi-sector* OR inter-sector* OR multisector* OR cross-sector* OR inter-department* OR interdepartment* OR transdisciplin* OR trans-disciplin*) NEAR/4 (approach* OR collab* OR partner* OR action* OR response*))) OR noft((environ* NEAR/5 (health* OR safety))) OR noft(((public* OR region* OR local* OR city OR cities OR municipal* OR provincial* OR province* OR territor* OR town* OR communit*) NEAR/5 (govern* OR health* OR policy OR policies)))) AND (noft(((Halifax OR Hamilton OR London OR Victoria OR Waterloo OR Welland OR Whitby OR Windsor OR Richmond OR Milton) NOT (UK OR Britain OR United Kingdom OR England OR Australia OR New Zealand))) OR noft(Abbotsford OR Ajax OR Aurora OR Barrie OR Belleville OR Blainville OR Brampton OR Brantford OR Brossard OR Burlington OR Burnaby OR Caledon OR Chatham Kent OR Chilliwack OR Clarington OR Coquitlam OR Drummondville OR Gatineau OR Granby OR Sudbury OR Guelph OR Halton Hills OR Kamloops OR Kawartha Lakes OR Kelowna OR Kingston OR Kitchener OR Langley OR Laval OR Levis OR Longueuil OR Maple Ridge OR Markham OR Mirabel OR Mississauga OR Montreal OR New Westminster OR Newmarket OR Niagara Falls OR Norfolk County OR North Bay OR North Vancouver OR Oakville OR Oshawa OR Ottawa OR Peterborough OR Pickering OR Port Coquitlam OR Prince George OR Quebec City OR Repentigny OR Richmond Hill OR Saanich OR Saguenay OR Saint-Hyacinthe OR Saint-Jean-sur-Richelieu OR Saint-Jerome OR Sarnia OR Sault Ste Marie OR Sherbrooke OR St Catharines OR Surrey OR Terrebonne OR Thunder Bay OR Toronto OR Trois-Rivieres OR Vancouver OR Vaughan) OR noft((British Columbia OR Colombie Britannique OR Ontario OR Quebec*)))

Results: 25
Dates: 2005-2026

Scopus

((TITLE-ABS-KEY ((public* OR region* OR local* OR city OR cities OR municipal* OR provincial* OR province* OR territor* OR town* OR communit*) W/5 (govern* OR health* OR policy OR policies))) OR (TITLE-ABS-KEY (environ* W/5 (health* OR safety))) OR (TITLE-ABS-KEY ((intersection* OR inter-section* OR intersector* OR multi-sector* OR inter-sector* OR multisector* OR cross-sector* OR inter-department* OR interdepartment* OR transdisciplin* OR trans-disciplin*) W/4 (approach* OR collab* OR partner* OR action* OR response*))) OR (TITLE-ABS-KEY (collab* W/4 (govern* OR action* OR approach* OR policy OR policies OR response*)))) AND ((TITLE-ABS-KEY (flood* OR hurricane* OR "polar amplification" OR "sea level rise*" OR "sea surface warming" OR "storm surge" OR "wild fire*" OR wildfire*)) OR (TITLE-ABS-KEY ((global* OR planet* OR earth) W/3 (heating OR warming))) OR (TITLE-ABS-KEY (heatshock* OR hyperthermia* OR "heat shock*" OR heat-shock)) OR (TITLE-ABS-KEY (thermal W/3 (expos* OR hazard*))) OR (TITLE-ABS-KEY ((increasing OR rising OR increase* OR extreme OR "rise in") W/3 (humidity OR humid OR temperature* OR weather* OR heat* OR hot* OR warm*))) OR (TITLE-ABS-KEY ("arctic amplification" OR "arctic shrinkage" OR "carbon footprint" OR "carbon offset*" OR "carbon sequestration" OR "carbon sink" OR "carbon sinks")) OR (TITLE-ABS-KEY (deglaciation OR desertification OR "natural disaster*" OR heatwave* OR drought OR droughts OR heat-dome*)) OR (TITLE-ABS-KEY ("global radiation*" OR "global temperature*" OR "global warming" OR "greenhouse effect" OR "greenhouse gas*" OR "heat wave*" OR heatwave* OR hurricane* OR "ice cap melt*" OR "ice mass loss*" OR "kyoto protocol" OR "landslide*" OR "land slide*" OR "mudslide*" OR "mud slide*" OR high-temp*)) OR (TITLE-ABS-KEY ("el nino" OR "la nina" OR "emissions reduc*" OR "emissions trading" OR flood*)) OR (TITLE-ABS-KEY ("paris accord" OR "permafrost melt*" OR "polar amplification" OR "polar ice melt*" OR "sea ice shrinkage" OR "sea level rise*" OR "sea surface warming" OR "storm surge*" OR "thermohaline circulation")) OR (TITLE-ABS-KEY (chlorofluorocarbon* W/2 (release OR concentration* OR atmosphere*))) OR (TITLE-ABS-KEY (climate W/3 (change* OR model?ing OR predict* OR resilience OR sensitivity OR action* OR variability OR adaptation OR crisis OR breakdown))) OR (TITLE-ABS-KEY ((glacial OR glacier) W/3 (retreat* OR melt*))) OR (TITLE-ABS-KEY ((extreme OR severe OR destructive) W/3 (temperature* OR weather* OR storm* OR hail OR wind* OR climate OR heat OR heating OR warm* OR humidity))) OR (TITLE-ABS-KEY (ozone W/2 hole*))) AND (((TITLE-ABS-KEY (halifax OR hamilton OR london OR victoria OR waterloo OR welland OR whitby OR windsor OR richmond OR milton) AND NOT TITLE-ABS-KEY (uk OR britain OR united AND kingdom OR england OR australia OR new AND zealand))

) OR (TITLE-ABS-KEY (british AND columbia OR colombie AND britannique OR ontario OR quebec*))) AND PUBYEAR > 2004 AND PUBYEAR < 2027

Results 830

GEOBASE

found in GeoRef for 1666-2024: ((((((((((((($extreme OR $severe OR $destructive) WN KY) AND ((temperature* OR weather* OR storm* OR $hail OR wind* OR $climate OR $heat OR $heating OR warm* OR $humidity) WN KY))) OR (((tornado* OR typhoon* OR wildfire* OR {wild fire*}) WN KY)) OR ((({Paris Accord} OR {permafrost melt*} OR {polar amplification} OR {polar ice melt*} OR {sea ice shrinkage} OR {sea level rise*} OR {sea surface warming} OR {storm surge*} OR {thermohaline circulation}) WN KY)) OR ((({global radiation*} OR {global temperature**} OR {global warming} OR {greenhouse effect} OR {greenhouse gas*} OR {heat wave**} OR heatwave* OR hurricane** OR {ice cap melt*} OR {ice mass loss*} OR {Kyoto Protocol} OR {landslide*} OR {land slide*} OR {mudslide*} OR {mud slide*} OR high-temp*) WN KY)) OR ((($El $Nino OR $La $Nina OR {emissions reduc*} OR {emissions trading} OR flood*) WN KY)) OR ((($deglaciation OR $desertification OR {natural disaster*} OR heatwave* OR $drought OR $droughts OR heat-dome*) WN KY)) OR ((((change* OR $modeling OR predict* OR $resilience OR $sensitivity OR action* OR $variability OR $adaptation OR $crisis OR $breakdown) WN KY) AND (($climate) WN KY))) OR (((($release OR concentration* OR atmosphere*) WN KY) AND ((chlorofluorocarbon*) WN KY))) OR ((({Arctic amplification} OR {Arctic shrinkage} OR {carbon footprint} OR {carbon offset*} OR {carbon sequestration} OR {carbon sink} OR {carbon sinks}) WN KY)) OR (((($increasing OR $rising OR increase* OR $extreme OR {rise in}) WN KY) AND (($humidity OR $humid OR temperature* OR weather* OR heat* OR hot* OR warm*) WN KY))) OR ((((stroke* OR hazard* OR expos* OR wave* OR $dome OR chronic* OR prolong* OR event* OR warn* OR shock* OR stress* OR alert* OR alarm*) WN KY) AND ((heat*) WN KY))) OR ((((expos* OR hazard*) WN KY) AND (($thermal) WN KY))) OR ((((global* OR planet* OR $earth) WN KY) AND (($heating OR $warming) WN KY))) OR ((((flood* OR hurricane* OR $polar $amplification OR $sea $level rise* OR $sea $surface $warming OR $storm $surge OR $wild fire* OR wildfire*.) WN KY) OR ((heatshock* OR hyperthermia* OR {heat shock*} OR $heat-shock) WN KY))) OR ((({ozone hole*}) WN KY)))) AND ((((((public* OR region* OR local* OR $city OR $cities OR municipal* OR provincial* OR province* OR territor* OR town* OR communit*) WN KY) AND ((govern* OR health* OR $policy OR $policies) WN KY))) OR ((((environ*) WN KY) AND ((health* OR $safety) WN KY))) OR ((((intersection* OR inter-section* OR intersector* OR multi-sector* OR inter-sector* OR multisector* OR cross-sector* OR inter-department* OR interdepartment* OR transdisciplin* OR trans-disciplin*) WN KY) AND ((approach* OR collab* OR partner* OR action* OR response*) WN KY))) OR ((((govern* OR action* OR approach* OR $policy OR $policies OR response*) WN KY) AND ((collab*) WN KY))))) AND ((((($Abbotsford OR $Ajax OR $Aurora OR $barrier OR $Belleville OR $Blainville OR $brayton OR $bradford OR $brossage OR $Burlington OR $burnable OR $caledonia OR $Chatham $Kent OR $Chilliwack OR $carrington OR $Coquitlam OR $Drummondville OR $Gatineau OR $grande OR $Sudbury OR $Guelph OR $Halton $Hills OR $Kamloops OR $karaha $Lakes OR $kanowna OR $Kingston OR $Kitchener OR $Langley OR $Laval OR $leaves OR $longueur OR $Maple $Ridge OR $Markham OR $mirage OR $Mississauga OR $Montreal OR $New $Westminster OR $newmark OR $Niagara $Falls OR $Norfolk $County OR $North $Bay OR $North $Vancouver OR $oroville OR $osaka OR $Ottawa OR $Peterborough OR $Pickering OR $Port $Coquitlam OR $Prince $George OR $Quebec $City OR $Repentigny OR $Richmond $Hill OR $Saanich OR $Saguenay OR $Saint-hyacinth OR $Saint-Jean-sur-Richelieu OR $Saint-Jerome OR $Sarnia OR $Sault $Ste $Marie OR $Sherbrooke OR $St $catherine OR $Surrey OR $Terrebonne OR $Thunder $Bay OR $Toronto OR $Trois-Rivieres OR $Vancouver OR $Vaughan) WN KY)) OR ((($British $Columbia OR $Colombie $Britannique OR $Ontario OR $Quebec) WN KY)))))) AND ((2026 OR 2025 OR 2024 OR 2023 OR 2022 OR 2021 OR 2020 OR 2019 OR 2018 OR 2017 OR 2016 OR 2015 OR 2014 OR 2013 OR 2012 OR 2011 OR 2010 OR 2009 OR 2008 OR 2007 OR 2006 OR 2005) WN YR)))) AND (({english} OR {french}) WN LA)))

Results: 1112

GEOREF

found in GeoRef for 1666-2024: ((((((((((($extreme OR $severe OR $destructive) WN KY) AND ((temperature* OR weather* OR storm* OR $hail OR wind* OR $climate OR $heat OR $heating OR warm* OR $humidity) WN KY))) OR (((tornado* OR typhoon* OR wildfire* OR {wild fire*}) WN KY)) OR ((({Paris Accord} OR {permafrost melt*} OR {polar amplification} OR {polar ice melt*} OR {sea ice shrinkage} OR {sea level rise*} OR {sea surface warming} OR {storm surge*} OR {thermohaline circulation}) WN KY)) OR ((({global radiation*} OR {global temperature**} OR {global warming} OR {greenhouse effect} OR {greenhouse gas*} OR {heat wave**} OR heatwave* OR hurricane** OR {ice cap melt*} OR {ice mass loss*} OR {Kyoto Protocol} OR {landslide*} OR {land slide*} OR {mudslide*} OR {mud slide*} OR high-temp*) WN KY)) OR ((($El $Nino OR $La $Nina OR {emissions reduc*} OR {emissions trading} OR flood*) WN KY)) OR ((($deglaciation OR $desertification OR {natural disaster*} OR heatwave* OR $drought OR $droughts OR heat-dome*) WN KY)) OR ((((change* OR $modeling OR predict* OR $resilience OR $sensitivity OR action* OR $variability OR $adaptation OR $crisis OR $breakdown) WN KY) AND (($climate) WN KY))) OR (((($release OR concentration* OR atmosphere*) WN KY) AND ((chlorofluorocarbon*) WN KY))) OR ((({Arctic amplification} OR {Arctic shrinkage} OR {carbon footprint} OR {carbon offset*} OR {carbon sequestration} OR {carbon sink} OR {carbon sinks}) WN KY)) OR (((($increasing OR $rising OR increase* OR $extreme OR {rise in}) WN KY) AND (($humidity OR $humid OR temperature* OR weather* OR heat* OR hot* OR warm*) WN KY))) OR ((((stroke* OR hazard* OR expos* OR wave* OR $dome OR chronic* OR prolong* OR event* OR warn* OR shock* OR stress* OR alert* OR alarm*) WN KY) AND ((heat*) WN KY))) OR ((((expos* OR hazard*) WN KY) AND (($thermal) WN KY))) OR ((((global* OR planet* OR $earth) WN KY) AND (($heating OR $warming) WN KY))) OR ((((flood* OR hurricane* OR $polar $amplification OR $sea $level rise* OR $sea $surface $warming OR $storm $surge OR $wild fire* OR wildfire*.) WN KY) OR ((heatshock* OR hyperthermia* OR {heat shock*} OR $heat-shock) WN KY))) OR ((({ozone hole*}) WN KY)))) AND ((((((public* OR region* OR local* OR $city OR $cities OR municipal* OR provincial* OR province* OR territor* OR town* OR communit*) WN KY) AND ((govern* OR health* OR $policy OR $policies) WN KY))) OR ((((environ*) WN KY) AND ((health* OR $safety) WN KY))) OR ((((intersection* OR inter-section* OR intersector* OR multi-sector* OR inter-sector* OR multisector* OR cross-sector* OR inter-department* OR interdepartment* OR transdisciplin* OR trans-disciplin*) WN KY) AND ((approach* OR collab* OR partner* OR action* OR response*) WN KY))) OR ((((govern* OR action* OR approach* OR $policy OR $policies OR response*) WN KY) AND ((collab*) WN KY))))) AND ((((($Abbotsford OR $Ajax OR $Aurora OR $barrier OR $Belleville OR $Blainville OR $brayton OR $bradford OR $brossage OR $Burlington OR $burnable OR $caledonia OR $Chatham $Kent OR $Chilliwack OR $carrington OR $Coquitlam OR $Drummondville OR $Gatineau OR $Granby OR $Sudbury OR $Guelph OR $Halton $Hills OR $Kamloops OR $karratha $Lakes OR $Kelowna OR $Kingston OR $Kitchener OR $Langley OR $Laval OR $leaves OR $longueur OR $Maple $Ridge OR $Markham OR $mirage OR $Mississauga OR $Montreal OR $New $Westminster OR $newmark OR $Niagara $Falls OR $Norfolk $County OR $North $Bay OR $North $Vancouver OR $oroville OR $osaka OR $Ottawa OR $Peterborough OR $Pickering OR $Port $Coquitlam OR $Prince $George OR $Quebec $City OR $Repentigny OR $Richmond $Hill OR $Saanich OR $Saguenay OR $Saint-hyacinth OR $Saint-Jean-sur-Richelieu OR $Saint-Jerome OR $Sarnia OR $Sault $Ste $Marie OR $Sherbrooke OR $St $catherine OR $Surrey OR $Terrebonne OR $Thunder $Bay OR $Toronto OR $Trois-Rivieres OR $Vancouver OR $Vaughan) WN KY)) OR ((($British $Columbia OR $Colombie $Britannique OR $Ontario OR $Quebec) WN KY)))))) AND ((2026 OR 2025 OR 2024 OR 2023 OR 2022 OR 2021 OR 2020 OR 2019 OR 2018 OR 2017 OR 2016 OR 2015 OR 2014 OR 2013 OR 2012 OR 2011 OR 2010 OR 2009 OR 2008 OR 2007 OR 2006 OR 2005) WN YR))))

Results: 975

EBSCO GreenFile

((Abbotsford or Ajax or Aurora or Barrie or Belleville or Blainville or Brampton or Brantford or Brossard or Burlington or Burnaby or Caledon or Chatham Kent or Chilliwack or Clarington or Coquitlam or Drummondville or Gatineau or Granby or Sudbury or Guelph or Halton Hills or Kamloops or Kawartha Lakes or Kelowna or Kingston or Kitchener or Langley or Laval or Levis or Longueuil or Maple Ridge or Markham or Mirabel or Mississauga or Montreal or New Westminster or Newmarket or Niagara Falls or Norfolk County or North Bay or North Vancouver or Oakville or Oshawa or Ottawa or Peterborough or Pickering or Port Coquitlam or Prince George or Quebec City

or Repentigny or Richmond Hill or Saanich or Saguenay or Saint-Hyacinthe or Saint-Jean-sur-Richelieu or Saint-Jerome or Sarnia or Sault Ste Marie or Sherbrooke or St Catharines or Surrey or Terrebonne or Thunder Bay or Toronto or Trois-Rivieres or Vancouver or Vaughan) OR (British Columbia or Colombie Britannique or Ontario or Quebec*) OR (((Halifax or Hamilton or London or Victoria or Waterloo or Welland or Whitby or Windsor or Richmond or Milton) NOT (UK or Britain or United Kingdom or England or Australia or New Zealand)))) AND ((((public* or region* or local* or city or cities or municipal* or provincial* or province* or territor* or town* or communit*) N5 (govern* or health* or policy or policies))) OR ((environ* N5 (health* or safety))) OR (((intersection* or inter-section* or intersector* or multi-sector* or inter-sector* or multisector* or cross-sector* or inter-department* or interdepartment* or transdisciplin* or trans-disciplin*) N4 (approach* or collab* or partner* or action* or response*))) OR ((collab* N4 (govern* or action* or approach* or policy or policies or response*)))) AND ((((global* or planet* or earth) N3 (heating or warming))) OR ((heat* N4 (stroke* or hazard* or expos* or wave* or dome or chronic* or prolong* or event* or warn* or shock* or stress* or alert* or alarm*))) OR ((thermal N3 (expos* or hazard*))) OR (((global* or planet* or earth) N3 (heating or warming))) OR (((increasing or rising or increase* or extreme or "rise in") N3 (humidity or humid or temperature* or weather* or heat* or hot* or warm*))) OR (("Arctic amplification" or "Arctic shrinkage" or "carbon footprint" or "carbon offset*" or "carbon sequestration" or "carbon sink" or "carbon sinks" or (chlorofluorocarbon* N2 (release or concentration* or atmosphere*)) or (climate N3 (change* or model?ing or predict* or resilience or sensitivity or action* or variability or adaptation or crisis or breakdown)) or deglaciation or desertification or "natural disaster*" or heatwave* or drought or droughts or heat-dome* or (earth N2 warming) or El Nino or La Nina or "emissions reduc*" or "emissions trading" or flood* or ((glacial or glacier) N3 (retreat* or melt*)) or "global radiation*" or "global temperature**" or "global warming" or "greenhouse effect" or "greenhouse gas*" or "heat wave**" or heatwave* or hurricane** or "ice cap melt*" or "ice mass loss*" or "Kyoto Protocol" or "landslide*" or "land slide*" or "mudslide*" or "mud slide*" or high-temp* or (ozone N2 hole) or "Paris Accord" or "permafrost melt*" or "polar amplification" or "polar ice melt*" or "sea ice shrinkage" or "sea level rise*" or "sea surface warming" or "storm surge*" or "thermohaline circulation" or ((extreme or severe or destructive) N3 (temperature* or weather* or storm* or hail or wind* or climate or heat or heating or warm* or humidity)) or tornado* or typhoon* or wildfire* or "wild fire*")))

2005-2026
Results 1658
