# Supplementary material for: Public health action on extreme heat in three Canadian provinces: a scoping review
Source: Public Health Rev. 2026 Jul 31;47:1609625. doi: 10.3389/phrs.2026.1609625 (PMC13473856; doi:10.3389/phrs.2026.1609625)
Supplement: Supplementary file 2 [file DataSheet2.pdf]

Supplementary Table 2a: Academic Literature Data Extraction Template Table

Note: A reduced version of the completed data extraction table of included academic articles is published in Supplementary File 4.

Level(s) of government	Jurisdiction(s)	Title	Author(s)	Publication year	Abstract	Study type	Aim/Purpose	Methods	Strategy or intervention	Key results & conclusions	Public health Role

Supplementary Table 2b: Grey Literature Data Extraction Template Table

Note: Completed data extraction tables of the reviewed grey literature are available upon request.

Level(s) of government	Organization	Title	Link	Date of publication	Result type	Authors	Public health role	Tool/Instrument (e.g., vulnerability assessment, legislation, regulation)
