# Supplementary material for: Public health action on extreme heat in three Canadian provinces: a scoping review
Source: Public Health Rev. 2026 Jul 31;47:1609625. doi: 10.3389/phrs.2026.1609625 (PMC13473856; doi:10.3389/phrs.2026.1609625)

Supplementary File 3: PRISMA diagram

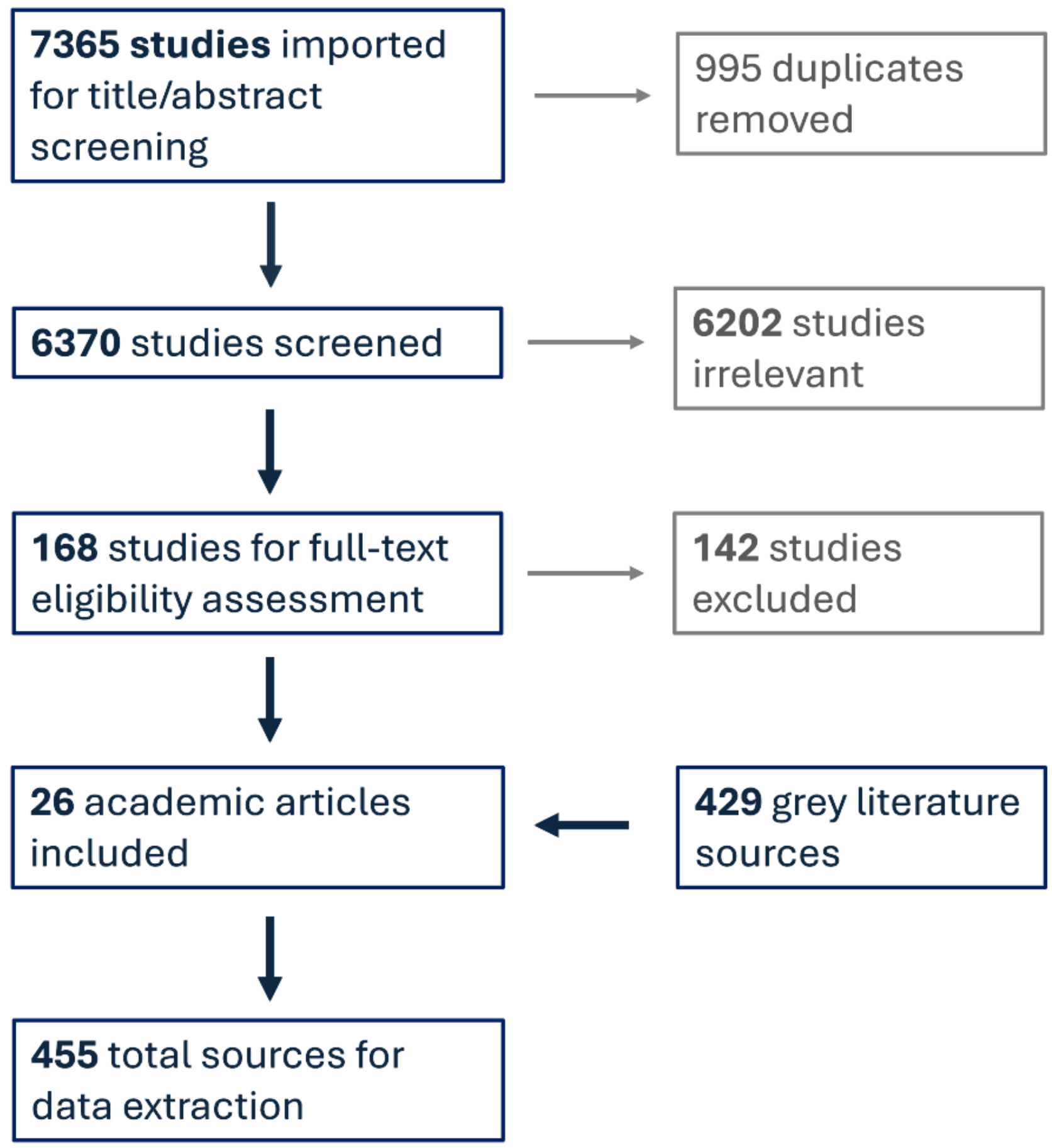

Supplement: Supplementary file 3 [file DataSheet3.pdf]
