# Supplementary material for: Public health action on extreme heat in three Canadian provinces: a scoping review
Source: Public Health Rev. 2026 Jul 31;47:1609625. doi: 10.3389/phrs.2026.1609625 (PMC13473856; doi:10.3389/phrs.2026.1609625)
Supplement: Supplementary file 4 [file DataSheet4.pdf]

Supplementary File 4: Included academic articles, by province

Jurisdiction(s) & Level(s) of Government	Title	Authors	Year	Study Type	Purpose	Strategy/ Initiative/ Intervention
**QUEBEC**						
Quebec (Provincial)	Transfer of climate knowledge via a regional climate-change management body to support vulnerability, impact assessments and adaptation measures	Vescovi, L; Bourque, A; Simonet, G; Musy, A	2009	Descriptive review of heat strategies or interventions	Describe how climate change knowledge transfer is conducted at Ouranos and provides two case studies in Quebec, with one focused on examining risk-assessment to public health due to extreme heat.	Risk assessment and knowledge transfer by the Ouranos consortium
Quebec (Provincial)	Research, impacts and adaptation in public health for the new climate of Quebec	Gosselin, P; Bélanger, D	2010	Descriptive review of heat strategies or interventions	Overview of the development of a public health research agenda on climate action in Quebec.	Research program for Ouranos and the Institut National de Santé Publique du Québec (INSPQ)
Quebec (Provincial)	The burgeoning field of transdisciplinary adaptation research in Quebec (1998–): a climate change-related public health narrative	Gosselin, P; Bélanger, D; Lapaige, V; Labbé ,Y	2011	Descriptive review of heat strategies or interventions	Describe how public health adaptation research is supporting transdisciplinary action and implementation, and how this has progressively been established.	Quebec Climate Change Action Plan

Quebec (Provincial)	An open source web application for the surveillance and prevention of the impacts on public health of extreme meteorological events: the SUPREME system.	Toutant, S; Gosselin, P; Belanger, D; Bustinza, R; Rivest, S	2011	Descriptive review of heat strategies or interventions	Develop a surveillance system for extreme weather events.	SUPREME: Système de surveillance et de prévention des impacts sanitaires des événements météorologiques extrêmes
Quebec (Provincial)	Health impacts of the July 2010 heat wave in Québec, Canada	Bustinza, R; Lebel, G; Gosselin, P; Bélanger, D; Chebana, F	2013	Epidemiological report	Estimates health impacts of the 2010 Quebec heat wave.	SUPREME
Quebec (Provincial)	A general and flexible methodology to define thresholds for heat health watch and warning systems, applied to the province of Québec (Canada)	Chebana, F; Martel, B; Gosselin, P; Giroux, JX; Ouarda, T	2013	Methodological development for establishing heat warning thresholds	Identifies appropriate heat health warning indicators as well as flexible and general thresholds that can be applied to a variety of regions and conditions.	Quebec heat health watch and warning systems (HHWWS)
Quebec, Montreal (Regional)	Implementation of the Montreal heat response plan during the 2010 heat wave.	Price, K; Perron, S; King, N	2013	Descriptive review of heat strategies or interventions	Describe Montreal's heat response plan and its application during the July 2010 heat wave.	Montreal Heat Response Plan
Quebec (Provincial)	An effective public health program to reduce urban heat islands in Québec, Canada	Beaudoin, M; Gosselin, P	2016	Descriptive review of heat strategies or interventions	Describe the implementation of urban heat island interventions in Quebec.	40 varied urban heat island interventions

Quebec, Montreal (Regional)	A Difference-in-Differences Approach to Assess the Effect of a Heat Action Plan on Heat-Related Mortality, and Differences in Effectiveness According to Sex, Age, and Socioeconomic Status (Montreal, Quebec).	Benmarhnia, T; Bailey, Z; Kaiser, D; Auger, N; King, N; Kaufman, JS	2016	Quantitative evaluation	Assess the effectiveness of the Montreal Heat Action Plan, implemented in 2004, on reducing mortality.	Montreal Heat Response Plan
Canada (Federal), Quebec (Provincial), Montreal & Estrie (Regional)	Intergovernmental relations for public health adaptation to climate change in the federalist states of Canada and Germany.	Austin, SE; Ford, JD; Berrang-Ford, L; Biesbroek, R; Tosun, J; Ross, NA	2018	Governance analysis	Examine how top-down and bottom-up intergovernmental dynamics patterned in different types of federal systems to enable public health adaptation at sub-national levels.	Public health adaptation collaborations
Quebec, Montreal (Regional)	The heterogeneity of vulnerability in public health: a heat wave action plan as a case study	Benmarhnia, T; Alexander, S; Price, K; Smargiassi, A; King, N; Kaufman, JS	2018	Qualitative evaluation	Critically examine how the concept of "vulnerability" is used in the Montreal heat response plan and how it is experienced by individuals diagnosed with schizophrenia and individuals with alcohol or drug addictions in the context of a heat response.	Montreal heat response plan
Quebec, Montreal (Regional)	The Montreal heat response plan: evaluation of its implementation towards healthcare	Price, K; Benmarhnia, T; Gaudet, J; Kaiser, D; Sadoine, ML;	2018	Qualitative evaluation	Assess the barriers and facilitators to implementation of the Montreal Heat Response Plan and evaluates the awareness of key elements of	Montreal Heat Response Plan

	professionals and vulnerable populations.	Perron, S; Smargiassi, A			the plan by healthcare professionals and individuals from vulnerable populations.	
Quebec (Provincial)	A heat-health watch and warning system with extended season and evolving thresholds	Issa, MA; Chebana, F; Masselot, P; Campagna, C; Lavigne, E; Gosselin, P; Ouarda, T	2021	Methodological development for establishing heat warning thresholds	Propose specific health-based monthly heat indicators and thresholds over an extended season from April to October in the northern hemisphere.	Quebec HHWWS
**ONTARIO**						
Ontario (Provincial & Municipal)	Adaptation to climate change in the Ontario public health sector	Paterson, J; Ford, J; Ford, L; Lesnikowski, A; Berry, P; Henderson, J; Heymann, J	2012	Descriptive review of heat strategies or interventions	Examine climate change adaptation in the public health sector in Ontario using information from interviews with government officials.	Climate change adaptation in the Ontario public health sector
Ontario (Municipal)	Development of a Climate Change Vulnerability Assessment Using a Public Health Lens to Determine Local Health Vulnerabilities: An Ontario Health Unit Experience	Levison, M; Butler, A; Rebellato, S; Armstrong, B; Whelan, M; Gardner, C	2018	Descriptive review of heat strategies or interventions	Describe the process of conducting a vulnerability assessment at a PHU, and the challenges, lessons, and future implications of conducting climate-focused vulnerability assessments within a PHU context.	Climate change vulnerability assessment

Ontario (Provincial)	Developing a harmonized heat warning and information system for Ontario: a case study in collaboration	Henderson, D; Aubin, L; Behan, K; Doyle, H; Gower, S; MacDonald, M; Mee, C; Richardson, G; Rochon, G; Shnabel, M; Storfer, J; Yagouti, A; Yusa, A	2020	Descriptive review of heat strategies or interventions	Outlines the process for establishing Ontario's harmonized system, including the impetus for action, role of key partners, and launch of the new system.	Ontario Harmonized Heat Warning & Information System (HHWIS)
Ontario (Municipal)	Developing and implementing a targeted health-focused climate communications campaign in Ontario—#MakeItBetter	Sanderson, M; Doyle, H; Walsh, P	2020	Descriptive review of heat strategies or interventions	Evaluates the effectiveness of a climate-related communication strategy and identify areas for future research.	Evidence-informed health-focused climate communication strategy
Ontario (Provincial)	Impact of Ontario's Harmonized Heat Warning and Information System on emergency department visits for heat-related illness in Ontario, Canada: a population-based time series analysis	Clemens, K; Ouédraogo, A; Le, B; Voogt, J; MacDonald, M; Stranberg, R; Yan, J; Krayenhoff, E; Gilliland, J; Forchuk, C; Van Uum, R; Shariff, S	2022	Quantitative evaluation	Evaluates the impact of Ontario's harmonized Heat Warning Information System on heat-related morbidity in urban regions; Explore whether effects differed across heat warning regions, and in various at-risk subpopulations.	Ontario HHWIS
**BRITISH COLUMBIA**						

British Columbia, Interior (Regional)	Development and implementation of a Heat Alert and Response System in rural British Columbia	Deegan, H; Green, J; El Kurdi, S; Allen, M; Pollock, S	2022	Descriptive review of heat strategies or interventions	Provides an overview of an ongoing cross-sectoral partnership within one rural BC community, located within the southern interior region, to develop and implement a Heat Alert and Response System.	BC Heat Alert and Response System (HARS)
British Columbia, Vancouver (Municipal)	Analysis of community deaths during the catastrophic 2021 heat dome	Henderson, S; McLean, K; Lee, M; Kosatsky, T	2022	Epidemiological report	Examines the effects of age, sex, neighbourhood deprivation, and surrounding environment on community deaths in Vancouver during the 2021 heat dome.	Public health response and policy intervention related to extreme heat events
British Columbia (Provincial)	A population-based case-control analysis of risk factors associated with mortality during the 2021 western North American heat dome: focus on chronic conditions and social vulnerability	McLean, KE; Lee, MJ; Coker, ES; Henderson, SB	2024	Epidemiological report	Identifies risk factors for death during the 2021 Extreme Heat Event by examining differences in chronic diseases and social vulnerability between the cases and controls.	Public health response and policy intervention related to extreme heat events
British Columbia (Provincial)	Hot Topic: A Systematic Review and Content Analysis of Heat-Related Messages During the 2021 Heat Dome in Canada	Tetzlaff, EJ; Goulet, N; Gorman, M; Richardson, GRA; Enright, PM; Meade, RD; Kenny, GP	2024	Content analysis	Examined the role of digital media in delivering heat-health messaging during an extreme heat event in Canada.	Public health messaging and guidance related to extreme heat events

British Columbia, Greater Vancouver (Regional)	Heat, smoke, and urban health: cooling and cleaner air centres as a tool for adaptation in a Canadian urban region	Apantaku, GO; Polgár, A; Giroux, F; Giang, A; Gladwin, D; Ellis, N	2025	Evaluation and governance analysis	Investigates the governance and effectiveness of cooling and cleaner air centres in the context of escalating heat and wildfire smoke hazards.	Cooling centres
**MULTI-PROVINCE**						
Quebec, Montreal (Regional)<br><br>Ontario, Toronto (Municipal)	How Toronto and Montreal (Canada) respond to heat	Kosatsky, T; King, N; Henry, B	2005	Descriptive review of heat strategies or interventions	Describes the Montreal and Toronto responses to extreme heat.	Montreal Heat Response Plan & Toronto's Hot Weather Response Plan
British Columbia (Provincial & Regional)<br><br>Ontario (Provincial & Regional)<br><br>Quebec (Provincial and Regional)	Heat-health messaging in Canada: A review and content analysis of public health authority webpages and resources	Tetzlaff, EJ; Wagar, KE; Johnson, SJI; Gorman, M; Kenny, GP	2025	Content analysis	Identifies what heat-health content is shared online by Canadian public health authorities and analyzes the material to develop an understanding of the content included within the resources.	Public health messaging and guidance related to extreme heat events

Quebec (Provincial), Montreal (Regional)<br><br>Ontario (Provincial), Toronto (Municipal)<br><br>British Columbia (Provincial), Vancouver (Regional)	A Comparative Case Study of Collaborative Governance for Intersectoral Extreme Heat Response in Vancouver, Toronto, and Montreal, Canada	Simpson, S; Seabrook, MSS; Di Ruggiero, E; Gautier, L; Miller, FA; Roerig, M; Xie, E; Allin, S	2026	Governance analysis	Examines the governance mechanisms shaping intersectoral extreme heat response in Vancouver, Toronto, and Montreal.	Quebec heat response plan; Montreal heat response strategies; Ontario Harmonized Heat Warning and Information System; Toronto Heat Relief Strategy; British Columbia Heat Alert and Response System; Vancouver Extreme Heat Initial Response Guideline
